# The early life immune dynamics and cellular drivers at single-cell resolution in lamb forestomachs and abomasum

**DOI:** 10.1186/s40104-023-00933-1

**Published:** 2023-10-12

**Authors:** Kailang Huang, Bin Yang, Zebang Xu, Hongwei Chen, Jiakun Wang

**Affiliations:** 1 Institute of Dairy Science, College of Animal Sciences, Zhejiang University, Hangzhou, 310058 China; 2 Key Laboratory of Molecular Animal Nutrition, Ministry of Education, Zhejiang University, Hangzhou, 310058 China

**Keywords:** Early life, Forestomachs, Four-chambered stomach, Immune cells, Immune system maturation, MIF signaling, Rumen, Ruminant development, Single-cell transcriptomic sequencing

## Abstract

**Background:**

Four-chambered stomach including the forestomachs (rumen, reticulum, and omasum) and abomasum allows ruminants convert plant fiber into high-quality animal products. The early development of this four-chambered stomach is crucial for the health and well-being of young ruminants, especially the immune development. However, the dynamics of immune development are poorly understood.

**Results:**

We investigated the early gene expression patterns across the four-chambered stomach in Hu sheep, at 5, 10, 15, and 25 days of age. We found that forestomachs share similar gene expression patterns, all four stomachs underwent widespread activation of both innate and adaptive immune responses from d 5 to 25, whereas the metabolic function were significantly downregulated with age. We constructed a cell landscape of the four-chambered stomach using single-cell sequencing. Integrating transcriptomic and single-cell transcriptomic analyses revealed that the immune-associated module hub genes were highly expressed in T cells, monocytes and macrophages, as well as the defense-associated module hub genes were highly expressed in endothelial cells in the four-stomach tissues. Moreover, the non-immune cells such as epithelial cells play key roles in immune maturation. Cell communication analysis predicted that in addition to immune cells, non-immune cells recruit immune cells through macrophage migration inhibitory factor signaling in the forestomachs.

**Conclusions:**

Our results demonstrate that the immune and defense responses of four stomachs are quickly developing with age in lamb's early life. We also identified the gene expression patterns and functional cells associated with immune development. Additionally, we identified some key receptors and signaling involved in immune regulation. These results help to understand the early life immune development at single-cell resolution, which has implications to develop nutritional manipulation and health management strategies based on specific targets including key receptors and signaling pathways.

**Supplementary Information:**

The online version contains supplementary material available at 10.1186/s40104-023-00933-1.

## Background

With the functional cooperation of a four-chambered stomach including the forestomachs (rumen, reticulum, and omasum) and abomasum, ruminants can convert low-quality forage into products that are important sources of high-quality animal proteins [1]. Therefore, a well-developed four-chambered stomach is essential for digestive tract function.

The structural development of the four stomachs and their micro-niches, including the microbiota and their metabolites, has received considerable attention, especially in the rumen [2–4]. However, the gene expression and cells involved in the immune functions of the four-chambered stomach remain largely unknown. It is widely accepted that immune functions are performed principally in specialized cells, notably immune cells. While non-immune cells, such as the epithelial and endothelial cells, play key roles in the regulation of the immune responses [5–7]. Epithelial cells are the first and most abundant cells exposed to microbial products, and play a critical role in initiating the immune responses in the mucosa of the gastrointestinal tract [8]. Young ruminants with developing four stomachs and a large influx of microbiota and their metabolites are highly susceptible to enteropathogenic diseases [9, 10]. The development of immune function in the four stomachs is crucial for the establishment of ecosystem homeostasis, which ensures optimal digestive function.

Genes enriched in immune response are associated with the microbial alpha diversity index of the rumen at early developmental stages [11]. However, the corresponding information about the reticulum, omasum, and abomasum is limited. The rumen, reticulum, and omasum have similar gene expression patterns, based on the analysis of 50 sheep tissues [12]; however, it remains unclear whether these stomachs have similar or different gene expression dynamics during early development. In particular, there are different cell subpopulations in different layers of the four-chambered stomach mucosa [13, 14], and the immune system is a highly complex and dynamic cellular and intercellular network [15]. Therefore, we hypothesized that the forestomachs might have different gene expression patterns related to the immune responses in early life with their specific cells. Single-cell RNA sequencing (scRNA-seq) provides an opportunity to assay gene expression at high resolution and construct immune development maps of complex organs [16]. To test our hypothesis, we investigated the dynamics of immune development during early age using transcriptomic analysis and integrated it with scRNA-seq analysis to illustrate the maps of immune function-related cells.

## Methods

### Sampling and transcriptomic sequencing

Three healthy male Hu sheep lambs were selected on d 5, 10, 15, and 25 from a commercial breeding farm cohort. The lambs were group raised in wooden pens with their mothers with a slotted floor under the exactly same conditions, and had free access to water and pellet feed (49.73% corn, 26.82% soybean meal, 20.23% wheat bran, 0.64% NaCl, 1.58% calcium hydrogen phosphate and 1.00% premix containing Fe, Zn, Cu, Mn, Co, I, Se, VA, VD and VE, dry matter basis). Before slaughter, we administered an intramuscular injection (0.001 mL/kg body weight) of Lumianning (Hua Mu, Changchun, China), a common anesthetic drug used in goat and sheep studies [17, 18], to minimize suffering of lambs during sacrifice. After complete loss of consciousness, as indicated by lying down with tongue extension and salivation, the lambs were sacrificed for samples by exsanguination. Tissues of the rumen ventral sac, reticulum, omasum, and abomasum were removed from the muscle layer. The mucosa was stripped from the tissue on ice, rinsed with precooled sterilized PBS (4 °C, pH = 6.8), and then snap-frozen in liquid nitrogen until they were stored at −80 °C. Total RNA was extracted from the mucosa of four stomachs using a total RNA extraction kit (Aidlab Biotechnologies Co., Ltd., Beijing, China), according to the manufacturer's instructions. RNA concentration and purity were assessed using the RNA Nano 6000 Assay Kit of the Bioanalyzer 2100 system (Agilent Technologies, Santa Clara, CA, USA); RNA degradation and contamination were assessed using 1% agarose gel electrophoresis. A total amount of 1 μg RNA per sample was used as input material to generate sequencing libraries using NEBNext® UltraTM RNA Library Prep Kit for Illumina® (NEB, Ipswich, MA, USA), following the manufacturer's recommendations. After cluster generation, the library preparations were sequenced on an Illumina NovaSeq platform (Illumina, San Diego, CA, USA), and 150 bp paired-end reads were generated. Clean reads were obtained from the raw data by removing reads containing adapters, poly-N, and low-quality reads that did not pass the Illumina chastity filter using CASAVA (version 1.8, Illumina).

### Weighted correlation network analysis of differentially expressed genes

High-quality reads were aligned to the sheep reference genome (*Ovis aries* v3.1) using TopHat2 software (v2.0.13). The number of reads per gene was counted using HTSeq-count (v0.9.1) based on ENSEMBL *Ovis aries* gene annotation files (http://www.ensembl.org/info/data/ftp/index.html). Gene expression levels were calculated by normalizing the number of reads to counts per million mapped reads (CPM), using the following equation: CPM = (number of reads/total mapped reads per library) × 1,000,000. Genes with CPM ≥ 1 in at least one lamb within at least one age were considered as expressed genes. We analyzed the differentially expressed genes (DEGs) using edgeR by comparing different stomachs at the same age and the same stomach between adjacent ages (d 10 vs. d 5, d 15 vs. d 10, and d 25 vs. d 15) [19]. For each gene, a *P*-value was obtained based on a negative binomial distribution model. The *P*-value < 0.05 and |fold changes|> 2 were set as the threshold to define DEGs. One DEG union set, including all the DEGs identified above, was used for the following analysis.

To identify the expression modules and their genes from the union set, we performed a Weighted Correlation Network Analysis (WGCNA) using the R package WGCNA [20]. Briefly, the CPM file of the DEG union set was used as the input. The output was the gene modules according to their expression patterns, and an optimal soft-thresholding power of six (Scale-free *R*^2^ = 0.86) was selected to ensure a scale-free topology. The module eigengene (MEs, the first principal component of the module) was chosen to represent the expression pattern.

### Tissue collection, dissociation, and single-cell RNA sequencing

Another 3 healthy male Hu sheep lambs at 25 days of age were purchased from the commercial breeding farm for stomach mucosa tissue collection and scRNA-Seq analysis. The methods used for animal treatment and tissue collection are described above. The mucosae were washed with an ice-cold washing buffer (D-Hank’s balanced salt solution containing 500 U/mL penicillin, 500 mg/mL streptomycin, 100 mg/mL gentamycin, and 5 mg/mL amphotericin B) until the solution was free of visible impurities and color. The tissues were transported to the laboratory in Dulbecco's modified Eagle's medium (DMEM) supplemented with 1,000 U/mL penicillin, 1,000 mg/mL streptomycin, 200 mg/mL gentamycin, and 10 mg/mL amphotericin B. The mucosae were dissociated according to a previously described method [21, 22] with some modifications. Briefly, mucosa tissues were cut into pieces (1 cm^2^), and 5 pieces of each stomach from each lamb were mixed separately to generate 4 mixed mucosa tissue samples for each stomach. After washing 5 times, the mucosa samples of the rumen, reticulum, and omasum were predigested with 0.25% trypsin–EDTA solution (Solarbio, Beijing, China) at 37 °C in a shaking warm-air bath. Every 10 min, the digestion solution was harvested and replaced with a fresh solution. The abomasum mucosa was digested with collagenase II (1.0 mg/mL; Sigma-Aldrich, St. Louis, MO, USA) for 2−4 cycles depending on the digestion status, with a 10-min incubation for each cycle [23]. The harvested solution was centrifuged at 300 × *g* for 5 min at 4 °C, and the digested solution was separated from the cell pellet. The dissociated cells were suspended in DMEM and filtered with a 40-µm nylon cell strainer (BD Falcon, San Jose, CA, USA). Cell viability was determined through trypan blue staining using a TC20 automated cell counter (Bio–Rad, Hercules, CA, USA). The ratio of viable cells was determined to exceed 70%. The number of cells in the single-cell suspension was counted using Countess (Thermo, Waltham, MA, USA) and adjusted to 700−1,200 cells/μL. The single cells in the cell suspension were captured, lysed, and their mRNA transcripts were ligated with barcoded indices at the 5'-end and reverse transcribed into cDNA using Chromium Controller and Chromium Single Cell 3' Reagent Kits (v2 chemistry CG00052; 10 × Genomics, Pleasanton, CA, USA), in accordance with the manufacturer’s protocol. The libraries were subjected to high-throughput sequencing on an Illumina NovaSeq PE150 platform, and 150-bp paired-end reads were generated by Novogene Bioinformatics Technology Co., Ltd. (Tianjing, China).

### Function enrichment analysis

Kyoto Encyclopedia of Genes and Genomes (KEGG) and Gene Ontology (GO) pathway enrichment analysis were performed using the “clusterProfiler” R package; a Benjamini–Hochberg corrected *P*-value < 0.05 was considered significant [24]. For GO pathway enrichment, only “Biological Process” GO terms were considered. Pathway activity in each sample was assessed using Gene Set Variation Analysis (GSVA) and Gene Set Enrichment Analysis (GSEA) using the GSVA R package and GSEABase R package, respectively [25, 26]. The immune associated KEGG pathways were selected to construct gene sets. The enrichment scores of pathways in all samples were calculated in GSVA analysis.

### Single cell data processing, cell clustering, and differential gene analysis

Reads were preliminarily filtered and processed using the Cell Ranger Single-Cell Software Suite (release 5.0.1) with default and recommended parameters. After the demultiplex raw data were generated using Illumina sequencers, the FASTQ files were aligned to the reference genome of *Ovis aries*. Only exonic reads that were uniquely mapped to the transcriptome were used for UMI counting. The 99^th^ percentile of the total UMI count divided by 10 was used as the cut-off for calling single cells [27]. The filtered single cells and their UMI count matrices were analyzed using the R package Seurat (version 4.0.0) [28]. Only genes expressed in more than three cells were retained, and each cell was required to express at least 200 genes. Furthermore, cells with > 20% of the genes from mitochondria and cells with doublets [29] were discarded. Library size normalization and scaling were performed using LogNormalize and ScaleData, respectively, and the results were log-transformed. Unsupervised clustering analysis was performed using the Seurat software. Highly variable genes were identified using the FindVariableFeatures function, and the average expression and dispersion of each gene were calculated. The number of significant principal components used for nonlinear dimensional reduction (t-SNE) analysis was chosen according to the elbow plot function, and cell clustering analysis was conducted using the FindClusters function.

### Cell–cell communication analysis

We identified the orthologous genes between mice and sheep using OrthoFinder (v.2.5.4) [30]. The orthologous genes were used to perform cell–cell communication analysis using R package CellChat [31]; the mouse data in CellChatDB database was selected. The cell types in different stomachs were investigated individually to determine the interaction networks.

### Cell concentration prediction in bulk RNA-seq data

To determine the dynamics of each cell type during the early developmental period in Hu sheep, we assessed the expression of marker genes for each cell type in bulk RNA-seq data (transcriptomic data of Hu sheep at 5, 10, 15, and 25 days of age) according to the method described by Lambrechts et al. [32] with slight modifications. We generated boxplots of the expression of each gene; it was log-normalized to an average expression of one in the sample at d 5 using the following equation: relative expression at one time point = (gene expression at one time point/gene expression at d 5). The marker genes for each cell type are listed in Additional file 1: Table S1. Data were analyzed using one-way analysis of variance with Tukey's multiple comparison test.

## Results

### Forestomachs share similar gene expression patterns during early developmental stages

**Fig. 1:**
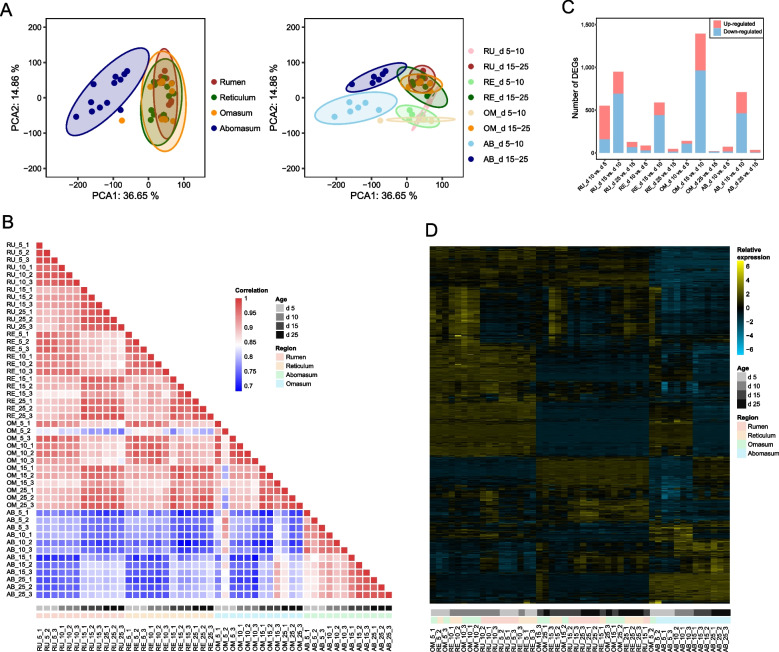
Gene expression patterns of four stomachs during early development. **A** Principal component analysis (PCA) of 48 samples across four ages and four stomachs based on the gene expression levels. The samples were clustered by tissue (left) and tissue plus age stage (right), respectively. **B** Heatmap showing the spearman correlation for 48 samples based on the gene expression level. **C** Bar plot showing the differentially expressed genes (DEGs) between adjacent age groups. **D** Heatmap showing the relative expression of all DEGs between adjacent age groups, rows (each representing a DEG) and columns (each representing one sample) are unsupervised clustered

To understand the mucosal gene expression patterns of four stomachs of ruminants at early ages, we collected 48 mucosal tissue samples from the rumen, reticulum, omasum, and abomasum, from each of the triplicate lambs at 5, 10, 15, and 25 days of age, and subsequently performed transcriptomic sequencing. After annotation and quality control, a total of 13,174 ± 448 genes (counts per million reads, CPM > 1) expressed in each sample were obtained. The gene expression pattern in abomasum clustered independently from that in forestomachs along the first principal component (PC1), accounting for 36.65% of the total variation (Fig. 1A). Spearman correlation analysis for all pairs of RNA-seq samples (Fig. 1B) further revealed the close similarities in all forestomach samples and the divergence between the abomasum and forestomachs. D 10 and 15 were the separatrices that divided the gene expression pattern in the same tissue into two closer stage blocks (d 5 to 10 and d 15 to 25).

Next, we determined the DEGs within the same tissue between any two adjacent ages (d 10 vs. d 5, d 15 vs. d 10, and d 25 vs. d 15). Stages from d 10 to 15 showed the highest number of DEGs in all tissues, followed by stages from d 5 to 10 and d 15 to 25 (Fig. 1C). Unsupervised hierarchical cluster analysis [33] across the ages of Hu sheep (Fig. 1D) showed that the DEGs could be clustered by both age and tissue type. The forestomachs and abomasum were classified into two clusters, and ages from d 5 to 10 and d 15 to 25 were classified into two clusters in the forestomachs and abomasum, respectively. Together, these selected DEGs provided a molecular signature for the developmental status during early developmental stages.

### Immune and defense responses in the mucosa of four stomachs are quickly developing with age during early life

**Fig. 2:**
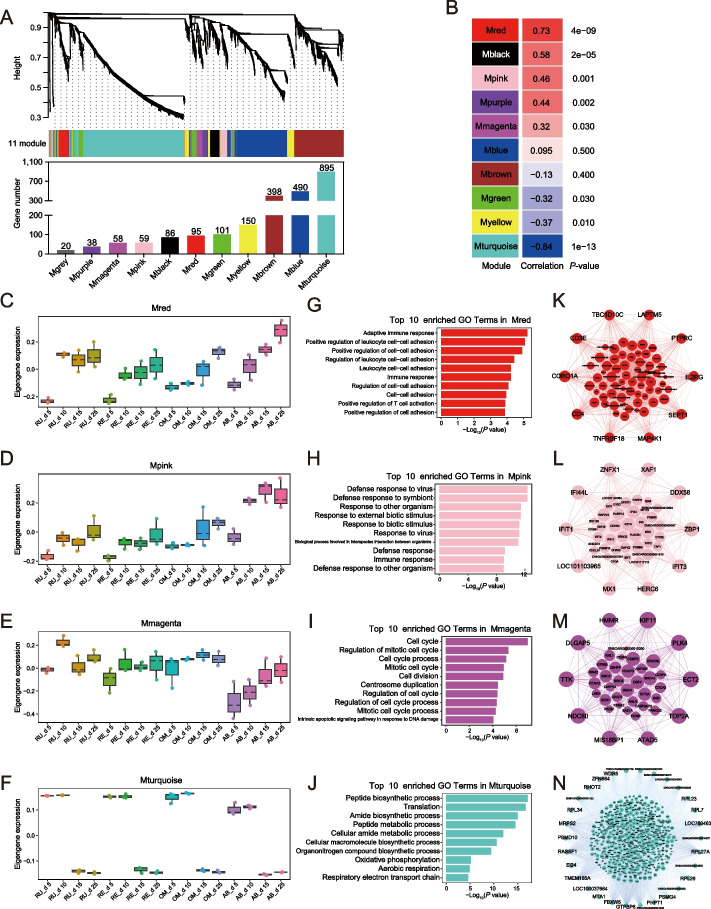
Gene co-expression network and their function in four stomachs during early development. **A** WGCNA cluster dendrogram and module assignment. Modules corresponding to branches are labeled with colors indicated by the color bands underneath the tree. The bar plot under the module assignment showing the gene number of each module. **B** Pearson correlation between modules and ages calculated based on the eigengene expression. **C–F** Boxplot showing the average eigengene expression of each tissue in Mred (**C**), Mpink (**D**), Mmagenta (**E**), and Mturquoise (**F**). **G–J** Top GO pathway enriched in Mred (**G**), Mpink (**H**), Mmagenta (**I**), and Mturquoise (**J**). Only GO terms for “Biological Process” were selected. **K–N** Co-expression network of Mred (**K**), Mpink (**L**), Mmagenta (**M**), and Mturquoise (**N**) constructed based on the WGCNA connection. Each module co-expression network were color-coded in module color. Each node represents one gene, the hub genes (top 10 in Mred, Mpink, and Mmagenta or top 30 in Mturquoise) were at the outermost of each networks

To characterize the dynamic changes in gene expression, we clustered all expression patterns using the WGCNA method. Except for the grey module that contained unclustered genes, we identified 10 main gene transcriptional modules, with the turquoise module (Mturquoise) containing the highest number of genes, followed by Mblue, Mbrown, and Myellow (Fig. 2A). We calculated the eigengene of each module and assessed module correlations with age using the module-eigengene. Five modules (Mred, Mblack, Mpink, Mpurple, and Mmagenta) were positively correlated with age, whereas three modules (Mgreen, Myellow, and Mturquoise) were negatively correlated with age (*P* < 0.05, Fig. 2B). GO and KEGG pathway enrichment showed that among the eight modules, no pathways were enriched in Mblack, Mpurple, Mgreen, or Myellow. Therefore, we focused on the four remaining age-associated modules, where eigengene expression of Mred and Mpink showed increased dynamics in each stomach (Fig. 2C–D), Mmagenta showed increased dynamics only in the abomasum (Fig. 2E), and Mturquoise showed a dramatic drop synchronously from d 10 to 15 in each stomach (Fig. 2F). Mred was associated with cell adhesion and its regulation, as well as immune function, such as “Adaptive immune response”, “Positive regulation of cell–cell adhesion”, “Positive regulation of T cell activation” (Fig. 2G), and “Th1 and Th2 cell differentiation”, “Th17 cell differentiation”, “Hematopoietic cell lineage”, “Cell adhesion molecules”, and “Cell adhesion molecules” (Additional file 2: Fig. S1A); Mpink was involved in defense response, and inflammatory or stimulus–response related pathways, respectively, such as “Defense response to virus” and “Defense response to symbiont” (Fig. 2H), and “RIG-I-like receptor signaling pathway”, “Toll-like receptor signaling pathway”, and “TNF signaling pathway” (Additional file 2: Fig. S1B); Mmagenta was associated with cell cycle, division, and their regulation, such as “Cell cycle”, “Regulation of mitotic cell cycle”, and “Cell division” (Fig. 2I), and “Homologous recombination” (Additional file 2: Fig. S1C); Mturquoise was enriched for peptide biosynthetic and metabolic process, and oxidative phosphorylation, such as “Peptide biosynthetic process”, “Translation”, and “Amide biosynthetic process” (Fig. 2J) and “Ribosome” and “Oxidative phosphorylation” (Additional file 2: Fig. S1D). The key genes driving the function of Mred included *CORO1A*, *CD3E*, *CD4*, *TBC1D10C*, *LAPTM5*, *IL2RG*, *MAP4K1*, *SEPT1*, *TNFRSF18*, and *PTPRC* (Fig. 2K); of Mpink, included *ZBP1*, *IFI44L*, *HERC6*, *DDX58*, *ZNFX1*, *XAF1*, *IFIT1*, *IFIT3*, *LOC101103965,* and *MX1* (Fig. 2L); of Mmagenta, included *KIF11*, *PLK4*, *ECT2*, *TOP2A*, *TTK*, *NDC80*, *DLGAP5*, *MIS18BP1*, *HMMR,* and *ATAD5* (Fig. 2M); and of Mturquoise, included ribosomal protein large subunit family genes (including *RPL23*, *RPL7*, *RPL27A*, *RPL26,* and *RPL34*), and *GTPBP6*, *TMEM185A*, *LOC780463*, *RASSF1*, *FBXW5*, *MRPS2*, *WDR5*, and *RHOT2* (Fig. 2N).

**Fig. 3:**
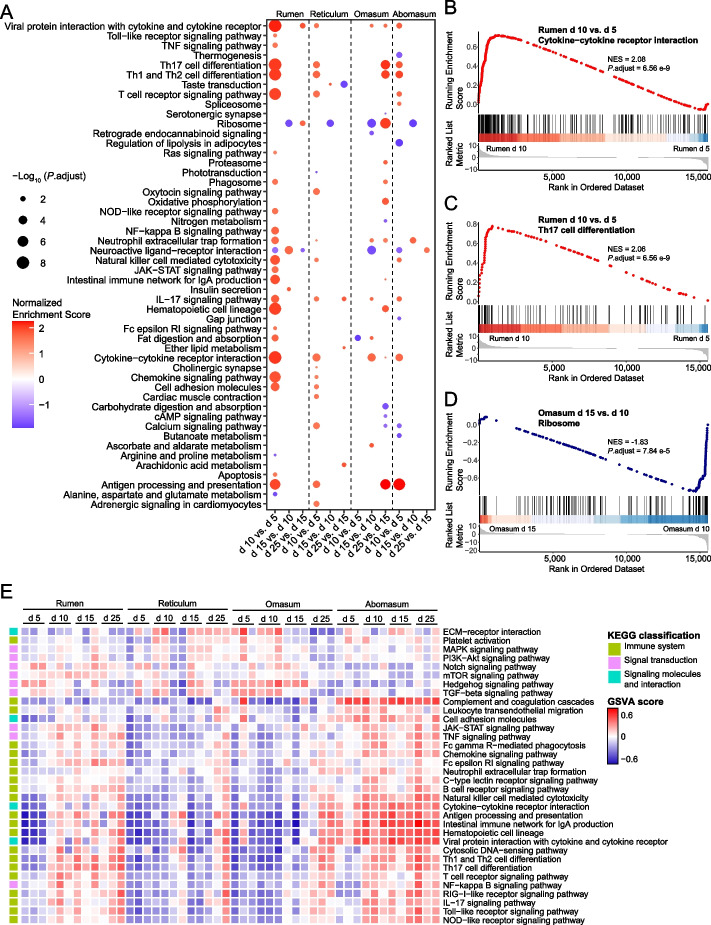
Function dynamics of four stomachs during early development. **A** Dot plots showing the activities of KEGG pathways enriched by get set enrichment analysis (GSEA) across the adjacent ages (adjusted *P* value < 0.05). **B–D** Examples of specific KEGG pathways of **A** including “Cytokine-cytokine receptor interaction” in rumen between d 10 vs. d 5 (**B**), “Th17 cell differentiation” in rumen between d 10 vs. d 5 (**C**), and “Ribosome” in omasum between d 15 vs. d 10 (**D**). **E** Heatmap showing immune associated pathway activities of each sample scored by gene set variation analysis (GSVA) across four ages

To validate the functional development of the four stomachs, we compared the functional differences using GSEA based on all expressed genes (CPM > 1). We identified 50 KEGG pathways exhibiting significant differential enrichment between adjacent ages and at least in one age stage in one stomach (Fig. 3A). Consistent with the functional enrichment and key genes shown in turquoise, the ribosome pathway was downregulated from d 10 to 15 in all stomachs (Fig. 3A and D), indicating downregulated protein synthesis [34]. However, most of the upregulated pathways were associated with immune function, such as cytokine-cytokine receptor interactions and Th17 cell differentiation, from d 5 to 10 in the rumen (Fig. 3B–C). To better understand dynamic immune function, we constructed an immune-associated gene set containing 34 pathways based on the KEGG database, and compared the activities of these pathways using GSVA. We observed a general increasing trend in the 34 immune-associated pathways from d 5 to 25, in all four stomachs (Fig. 3E). However, these pathways showed a delayed increase (d 15 or 25) in the reticulum and omasum compared to that in the rumen and abomasum (d 5 or 10). Taken together, the results from both GSEA and GSVA suggested a marked functional focus on the immunity and defense of the four stomachs during early sheep developmental stages.

### Immune cells and endothelial cells play essential roles in immune and defense function during early developmental stages

**Fig. 4:**
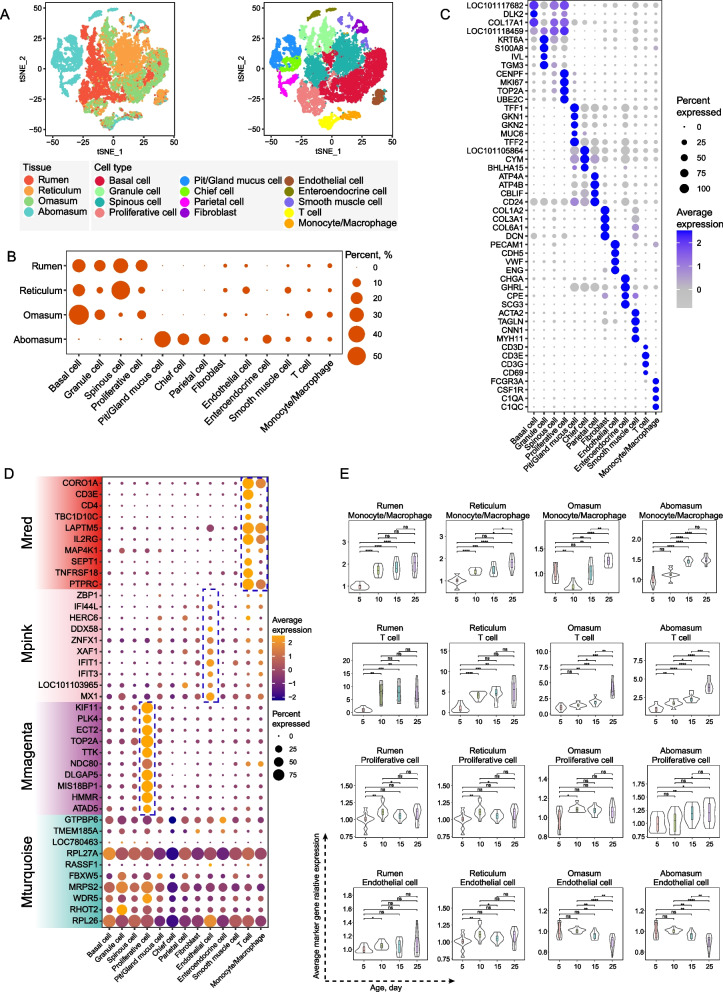
Single cell view and signature of four stomachs. **A** t**-**SNE analysis of 22,589 cells from rumen, reticulum, omasum, and abomasum at d 25, with four tissues labeled in left side, and 12 major cell types labeled in right side with different colors. **B** Dot plot showing the expression of marker genes for the cell types. **C** The proportion of each cell type among four different tissues. **D** Dot plot showing the expression of hub genes of Mred, Mpink, Mmagenta, and Mturquoise in 13 cell types; the hub genes of each module were identified through WGCNA analysis (see Fig. 2E). **E** Average expression of marker genes for monocytes/macrophages, T cells, proliferative cells, and endothelial cells of four tissues across four ages in the transcriptomic data, respectively. Data across four ages were analyzed using one-way analysis of variance with Tukey’s multiple-comparisons test (^*^P < 0.05, ^**^P < 0.01, ^***^P < 0.001, ^***^^*^P < 0.0001, ns: no significant)

Assessing the immune functions of various cell types is essential to fully elucidate the mechanisms underlying immune function development. In this study, we performed a single-cell sequencing analysis of cells dissociated from the entire mucosa of the rumen, reticulum, omasum, and abomasum of three Hu lambs at 25 days of age. Among these, 5,820, 6,096, 6,420, and 4,433 cells originated from the rumen, reticulum, omasum, and abomasum, respectively (Fig. 4A). We classified these cells into groups of cell types using graph-based clustering, based on informative principal components. Based on the expression of the marker genes (Fig. 4B, Additional file 1: Table S1), we classified these cells into known cell lineages: basal cells, granule cells, spinous cells, proliferative cells, pit/gland mucus cells, chief cells, parietal cells, fibroblasts, endothelial cells, smooth muscle cells, monocytes, macrophages, and T cells (Fig. 4B). The proportion of each cell lineage varied significantly among the four tissue types (Fig. 4C).

To further identify the major cell types dominating the maturation of immune and defense functions, we investigated the expression of the hub genes identified in Mred, Mpink, Mmagenta, and Mturquoise for each cell type (Fig. 4D). All of the Mred hub genes were highly expressed in T cells; among them, *CORO1A*, *LAPTM5*, *IL2RG*, *MAP4K1*, and *PTPRC* were highly expressed in both T cells and monocytes or macrophages. Eight Mpink hub genes (*IFI44L*, *DDX58*, *ZNFX1*, *XAF1*, *IFIT1*, *IFIT3*, *LOC101103965*, and *MX1*) were highly expressed in endothelial cells. Consistent with the module enrichment analysis results, all magenta hub genes were highly expressed in proliferative cells. However, the expression of the turquoise hub genes did not significantly differ among the different cell types. These results suggest that T cells, monocytes, or macrophages dominate Mred; endothelial cells and proliferative cells dominate Mpink and Mmagenta, respectively; and Mturquoise is dominated by multiple cell types that work together.

We assessed the average relative expression of marker genes (the marker genes were consistent with Fig. 4B) for monocytes/macrophages, T cells, and endothelial cells, in the transcriptomic data across the four time points. We observed an age-dependent increase in the expression of monocytes/macrophages and T cells marker genes in the four stomachs (Fig. 4E). There was an increase in only the proliferative cells from d 5 to 10 in forestomachs, and a continuous increase in proliferative cells in the abomasum, indicating the ratio dynamics of the above cell types from d 5 to 25. However, the expression of endothelial cell marker genes did not change over time in the rumen and reticulum, and it decreased from d 10 to 25 over time in omasum and abomasum. This phenomenon combined with the dynamic of Mpink hub gene expression indicated that the expression of Mpink hub gene of endothelial cells rather than the cell ratio increase with age.

The integration of single-cell results and functional enrichment analyses through GSVA and GSEA confirmed that the main events of development were age-dependent increases in immune cells and their immune functions, as well as in endothelial cells and their defense-related functions in the early developmental stages of Hu sheep.

### Widespread immunity activation occurs in different stomachs

**Fig. 5:**
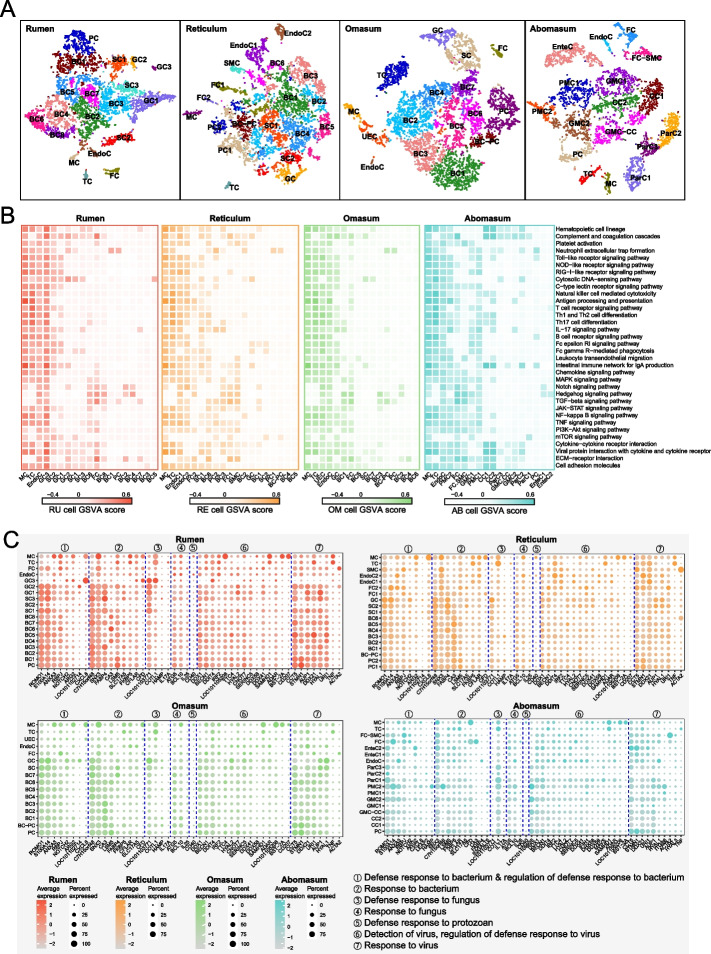
Widespread activation of immune function and their associated gene expression in different cell types in four stomachs. **A** t-SNE maps of the cells in rumen, reticulum, omasum, and abomasum (left to right). **B** Immune-associated pathway activities of each cell type in rumen, reticulum, omasum, and abomasum (left to right). **C** Dot plot showing the expression patterns of genes associated with the defense or response to microorganism in different cells in rumen (upper left), reticulum (upper right), omasum (lower left), and abomasum (lower right). The genes and their function were obtained from GO database, only genes that expressed in at least one cell type (Percent expressed > 0) were kept. The names of the GO terms were used as the summary of the function of these genes (①–⑦)

In addition to classical immune cells, non-immune cells play an important role in the immune function of different tissues [35]. To determine whether non-immune cells in four stomach tissues are involved in immune functions, we systematically investigated the global involvement of all cell types in individual tissue immunity. Based on the known cell markers (Additional file 2: Fig. S2A–D; Additional file 1: Table S2–S5), we defined 19, 19, 16, and 18 clusters of rumen, reticulum, omasum, and abomasum cells, respectively (Fig. 5A). The forestomachs share similar cell types including basal cells (BC), spinous cells (SC), granule cells (GC), proliferative cells (PC), fibroblasts (FC), endothelial cells (EndoC), T cells (TC) and monocytes/macrophages (MC). In addition, a type of smooth muscle cells (SMC) in reticulum and undefined epithelial cells (UEC) in omasum were identified. In addition to PC, FC, EndoC, TC and MC, the diverse cell types including parietal cells (ParC), chief cells (CC), pit mucus cells (PMC), gland mucus cells (GMC) and enteroendocrine cells (EnteC) were identified in abomasum.

We performed a cross-tissue comparison of all cell types for immune- and defense-related pathway enrichment using GSVA. In addition to immune cells (TC and MC) in all four tissues, some non-immune cell types had high immune activities, particularly EndoC, in the four stomachs. In addition, GC3 in the rumen, FC2 and SC1 in the reticulum, UEC and GC in the omasum, and PMC2 and FC in the abomasum exhibited relatively high immune activity (Fig. 5B). These results suggest that non-immune cells undergo a layer of cellular regulation for tissue-specific immunity. Among these immune-related pathways, the toll-like receptor and NOD-like signaling receptor signaling pathways exhibit distinct activities among different cell types due to their important roles in both innate and adaptive immune activation [36, 37]. Although immune cells are central to pattern recognition receptor (PRR) signaling responses, many other cell types, including epithelial, stromal, and endothelial cells, express PRRs [36], which recognize a broad range of microbial-associated molecular pattern ligands and trigger immune activation. Therefore, we further explored the expression of PRRs, including that of toll-like receptors (TLRs), NOD-like receptors (NLRs), and RIG-I-like receptors (RLRs), in all cell types across the four stomach tissues (Additional file 2: Fig. S3A–D). Notably, there is a distinct group of non-immune cells, including GC3 in the rumen, EndoC1 in the reticulum, FC in the omasum, and EndoC in the abomasum, that show high expression of PRR genes. We also investigated the expression of G protein-coupled receptors (GPCRs) that mediate most of the physiological responses to various signaling molecules including microbial metabolites, thus involved in regulating gastrointestinal mucosal immunity and maintaining intestinal barrier (Additional file 2: Fig. S4A–D). Here we focused on fatty acid receptors because they are most studied and abundant group of microbial metabolites, we also focused on the members of GPCR families that have been validated to be activated by microbiome cultures in a recent high-throughput screening study [38]. The GPCR families shared similar expression patterns between the mucosa of forestomachs, compares to those in abomasum. For example, the free fatty acid receptor 2 (*FFAR2*, also known as *GPR43*) and free fatty acid receptor 3 (*FFAR3*, also known as *GPR41*) that sense short-chain fatty acids (SCFAs), were lowly expressed in all cell types, whereas the *FFAR4* (also known as *GPR120*) that senses medium- and long-chain unsaturated fatty acids, was highly expressed in the GC and SC and some subtypes of BC in forestomachs. In addition, fatty acid binding protein 4 (*FABP4*) and 5 (*FABP5*) were highly expressed in most of PC, BC, SC and GC subtypes in forestomachs. However, *FABP3* was highly expressed in ParC in abomasum. Although succinate receptor (*SUCNR1*), adrenergic receptors (ADRA family), cholinergic receptors (CHRM family), dopamine receptors (DRD family), histamine (HRH family) and 5-hydroxytryptamine receptors (HTR family) can be activated by multiple microbial strains culture [38], most genes coding for these families were lowly expressed. For example, *SUCNR1* that can be activated by various microbial culture including the strains belong to *Bacteroides*, *Prevotalla*, *Escherichia coli*, were not nearly expressed in the all cell subtypes in forestomachs and abomasum. We also noted that *F2RL1*, *HTRA1*, *HTRA2* were highly expressed in the most of PC, BC, SC, GC, FC and EndoC subtypes in forestomachs.

In addition to the differences in the receptors that recognize microorganisms and their metabolites, the key responses of different cells to various microorganisms remain undefined. Therefore, we analyzed the expression of genes related to host defense and responses to bacteria, fungi, protozoans, and viruses, based on the GO database (Fig. 5C). Genes related to defense and response to bacteria were highly expressed in epithelial cells, including PC, BC, SC, and GC in the forestomachs; for example, *ROMO1*, which encodes a protein with antimicrobial activity against a variety of bacteria [39] and *S100A14*, one of the S100A gene families associated with the immune system [40]. Genes related to defense against fungi were highly expressed in the SC and GC; this includes *LOC101103771* (also known as *S100A12*), which exhibits antifungal activity through zinc sequestration [41, 42]. However, the *CD40* that defense to protozoan was lowly expressed in all cell types. Genes related to detection, response, and regulation of defense responses to viruses were highly expressed in a wide range of cell types. However, genes related to the responses to microbes, including bacteria, fungi, protozoans, and viruses, were expressed in a cell type-specific fashion in abomasum cells; for instance, S100A14 was highly expressed only in PMC2 and FC. Collectively, we identified cell types with expression of specific genes associated with the host responses to various microorganisms.

### Non-immune cells recruit immune cells through MIF signaling

**Fig. 6:**
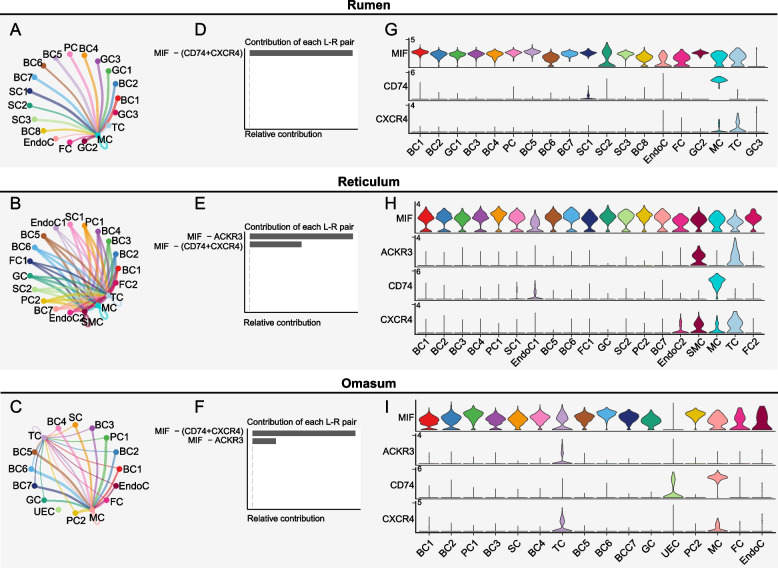
Communication between non-immune cells and immune cells. **A–C** MIF signaling networks in rumen (**A**), reticulum (**B**), and omasum (**C**), respectively. In the networks, edge width represents the communication probability. **D–F** The bar plot showing the relative contribution of each ligand-receptor pair to the overall communication MIF signaling networks in the rumen (**D**), reticulum (**E**), and omasum (**F**), respectively. **G–I** Violin plots showing the expression distribution of ligand and receptor genes of MIF signaling in different cell types, in the rumen (**G**), reticulum (**H**), and omasum (**I**), respectively

To understand the communication between immune and non-immune cells in coordinating immune responses, we used CellChat to construct ligand-receptor maps. Notably, our analysis revealed that T cells and monocytes/macrophages interacted with non-immune cells through the macrophage migration inhibitory factor (MIF) signaling pathway in the rumen, reticulum, and omasum (Fig. 6A–C). Contribution analysis of ligand-receptor pairs identified that MIF served as the ligand, with CD74/CXCR4 or ACKR3 serving as the receptors (Fig. 6D–F). Notably, the MIF-(CD74/CXCR4) ligand-receptor pair exhibited the highest contribution to MIF signaling in the rumen and omasum (Fig. 6D and F), while MIF-ACKR3 ligand-receptor pair showed the highest contribution in the reticulum (Fig. 6E). Furthermore, we investigated the expression levels of these ligands and receptors in each cell subtype of the forestomach mucosa. Except for granular cell 3 in the rumen and undefined epithelial cells in the omasum, all other cell subtypes expressed high levels of MIF (Fig. 6G–I). Monocytes/macrophages are the major source of CD74, whereas T cells are the major source of CXCR4 and ACKR3. Together, our results revealed that forestomach cells showed a generally high level of MIF, which potentially recruits immune cells into the tissue.

## Discussion

Early life represents a period of unique immune development, during which the foundation for lifelong immunity is laid [43]. The microbes living in the gastrointestinal tract play critical roles in the development of organized lymphoid structures and in the function of immune cells [44]. The surfaces of ruminants’ forestomachs are continuously exposed an enormous microbial load from both contents and walls themselves; however, the presence of immune cells in these stomachs remains a speculative issue [45–47], and there is even less research on the development of the immune system in ruminant forestomachs. In this study, by combining transcriptomic sequencing and single-cell transcriptomic sequencing analysis, we present a wide gene expression dynamic across four stomach entire mucosa and time points, and a comprehensive catalog of immune-related functions across the four stomachs entire mucosa at single-cell resolution, we also highlight the intercellular communication between non-immune cells and immune cells. These new findings will contribute to understanding the window of opportunity for modulation of the gastrointestinal tract, not only in terms of four-chambered stomach development, but also in forestomachs and abomasum-related disorders during early life.

### The early window of immune system development might be a good opportunity for regulation

The early life represents a critical window of immune development during which the foundation for lifelong immunity is laid. During the first postnatal day of human, a strong systemic immune response occurs in infants, which is dominated by innate immune cells such as monocytes and elevated circulating cytokines [48]. Similar to human, the current study identified that two gene co-expressed modules involved in immune activation and defense were positively correlated with age in four stomachs, which was consistent with previous studies that reported immune development in the goat rumen during early life [11]. Activated immune responses of forestomachs might be associated with lower susceptibility to subacute ruminal acidosis (one of the metabolic ruminant disorders induced by high-concentrate diets), as evidenced by the higher expression of toll-like receptor 2 (*TLR2*) and toll-like receptor 4 (*TLR4*) in the rumen mucosal epithelium, compared to that in the mucosal epithelium of acidosis-susceptible cattle [49]. Therefore, they are of great significance for the improvement of resistance to subacute ruminal acidosis by strengthening the immune response.

### Atlas of immune-related function among different cells provide precise strategies for regulation

Single-cell transcriptomic analyses of the rumen revealed major and novel cell types [13, 14, 50, 51]. Cell types and their functional similarities in the rumen, reticulum, and omasum have been investigated [13]. However, the early life immune function development in the forestomachs remains largely unknown, in particular the cellular heterogeneity of immune function. Integrating transcriptome and single-cell transcriptome data, we connected the age-related gene modules and their hub genes with cell types in four stomachs simultaneously. These hub genes with specific function facilitate to identify major cell types that mediate the function of modules in early life. For example, red module hub genes that were highly expressed in T cells, monocytes, and macrophages are related to immune system or cell signaling regulation. These hub genes including T cells marker gene *CD4* and *CD3E*, additionally, among other hub genes, *IL2RG* encodes the interleukin 2 receptor subunit gamma chain that serves as a part of several important cytokines; *TNFRSF18* encodes a member of TNF-receptor superfamily, which is upregulated upon T-cell activation; *PTPRC* encodes a member of the protein tyrosine phosphatase family, which is an essential regulator of T- and B-cell antigen receptor signaling; *LAPTM5* encodes a transmembrane receptor that is associated with lysosomes; other hub genes including *MAP4K1*, *CORO1A*, and *TBC1D10C* mediate signal transduction events that play a role in the regulation of cell development, activation, growth, and motility. The pink module hub genes, which were highly expressed in endothelial cells, are related to the innate immune response. For example, *ZBP1* induces type-I interferon production; *IFI44L*, *IFIT1*, *IFIT3*, *and MX1* (also known as *IFI78*) are interferon-induced proteins involved in the defense responses against viruses; *DDX58* (also known as *RIG-I,* coding for one type of pattern recognition receptor) is involved in viral RNA recognition and regulation of the antiviral innate immune response. Considering the critical role of these hub genes, our results indicated that the function of red module was dominant in T cells, monocytes, and macrophages. This was further verified by the increased average marker gene expression of T cells and macrophages with age in bulk sequencing analysis. The function of pink module was dominated by endothelial cells, which form a barrier between vessels and tissues [52], and have an essential paracrine function through the secretion of chemokines, interleukins, interferons, and growth factors, thus coordinating the immune responses [53].

The forestomachs tissue from the luminal to the innermost were composed of mucosal epithelial layer, submucosal layer and muscle layer, respectively. In addition, the epithelia of forestomachs are stratified squamous mucosal epithelium, which is composed of four distinct strata (from outside to inside are corneum, granulosum, spinosum, and basale), whereas the abomasum is columnar epithelium [1]. In the current study, we focus on mucosa of four stomachs (Additional file 2: Fig. S5), which might be the reason of the existence of immune cells, fibroblasts, endothelial cells and other non-epithelial cells. These non-epithelial cells play important roles in maintain of epithelium structure. For example, fibroblasts produce extracellular matrix to support the structure of mucosal epithelium [54]. The smooth muscle cells were identified in reticulum in our results, which is consistent with the histomorphology of the reticulum, where the muscularis mucosae is present in the primary reticular crest of reticulum [55]. However, although recent studies have revealed the cell types of the rumen or four stomachs mucosa using scRNA-seq analysis [13, 14, 50, 51], the spatial resolution are limited. Future studies should strive to standardize the sample and cell dissociation methods for the comparison of different studies. In addition, spatial omics should be integrated to analysis, to better understand the locations of cell types, functions and their interactions.

Although various microbes attach to the corneum of rumen mucosal epithelium, it have long been considered that these microbes remain unable to penetrate the stratum granulosum due to the protective barrier of the corneum and mechanical strengthening of the mucosal epithelium by tight junctions (occludins and claudins), adherin junctions, and desmosomes of the granulosum [56, 57]. However, our study showed that genes associated with host defense or response to microorganisms were widely expressed in different cell types, including basal cells in the basal layer and spinous cells in the spinosum layer. This may be attributed to the fact that, in addition to the microbiota itself, the components and metabolites of the microbiota can trigger defensive responses from both epithelial cells and the immune system [5].

Pattern-recognition receptors (PRRs) provide distinct pathways for the recognition of microbial ligands or endogenous signals associated with pathogenesis [58]. In the current study, members of the Toll-like receptor (TLR), NOD-like receptor (NLR), and RIG-I-like receptor (RLR) family genes are highly expressed in the non-immune cells. For example, *TLR4* that widespread expressed in various cell subtypes in forestomachs was reported to senses lipopolysaccharides (LPS) [59]. Previous studies have suggested that the translocation of ruminal LPS can trigger a local innate immune response when epithelial barrier function was disrupt [60]; however, in vitro repeated LPS stimulation could induce ruminal epithelial cells produce a tolerogenic effect [47]. Thus, widespread expressed *TLR4* gene in the early life might indicate the establishment of immune tolerance. Free fatty acid receptors have attracted widespread attention, because of the great abundance of free fatty acids and their importance in mediating the immune and growth. Previous study reported that *FFAR3* (also known as *GPR41*) mediated the regulation of SCFAs on the genes involved in immune cell recruitment and epithelial immune barrier in bovine rumen epithelial cells [61], in addition, both *FFAR3* and *FFAR2* (also known as *GPR43*) can be detected in rumen mucosal epithelium although *FFAR2* exhibited lower level [62]. However, we and another study [63] found that both *FFAR2* and *FFAR3* were little expressed in the rumen mucosal epithelium. This inconsistent result might be due to the disturbances of unmapped reads in transcriptome assembling, further identification including at protein level are required. Free fatty acids receptor 4 (*FFAR4*, also known as *GPR120*), a sensor for long-chain fatty acids, has been reported to mediate ant-inflammatory and insulin-sensitizing activity [64] as well as modulate food intake and body weight gain in mouse and human [65]. Interestingly, *FFAR4* was highly expressed in the forestomachs in the current study, which provide a candidate molecular target for both immune and growth regulation in ruminant based on the microbiota or their metabolites.

The immune responses to different types of microorganisms are cell type-specific, and it is worth noting that the genes associated with the defense response against bacteria are highly expressed in basal cells, spinous cells, and granule cells; however, the genes associated with the defense responses against fungi are highly expressed in granule cells, which are located at the outermost layer of the mucosal epithelium. These results suggest a spatial organization of the host defense responses to microorganisms. The outcome of microbiota colonization with select taxa likely depends on both the characteristics of the colonizing species and their site-specific ability to interact with the host [59, 66]. Further investigations are necessary to obtain a clearer understanding of the immune responses against various microorganisms in four stomachs.

### MIF signaling pathway is a potential route for early immune regulation in the forestomachs

Communications between epithelial cells and the resident immune cells are crucial for maintaining homeostasis and coordinating appropriate responses to disease. These communications can occur through cell-to-cell contact or by the release or recognition of soluble mediators [67]. By investigating the communication among cell types in the stomach, we identified a role for non-immune cells in recruiting T cells, monocytes, and macrophages in the forestomachs via MIF signaling. Here we found that MIF produced by non-immune cells plays an important role in recruiting immune cells, which express high levels of CD74 and CXCR4 simultaneously or ACKR3 in response to MIF. Previous studies reported that through the binding and activation of these receptors, MIF signaling drives numerous inflammatory and malignant diseases [68], and the downstream pathways involved in sustained ERK-1/2 activation [69, 70], regulation of JAB1 [71], and p53 transcriptional activity [69], which reflect a wider function of growth regulation, apoptosis, and cell cycle control. However, the mechanisms underlying the involvement of MIF signaling in the forestomachs remain largely unexplored. Unraveling the complexity of MIF signaling remains an exciting area for the basic investigation of the interaction between non-immune cells and immune cells.

### Limitations of the study

The current study remains several limitations. First, it is difficult for us to further distinguish immune cell types including dendritic cells, various subtypes of T cells, due to the limitation of cell number. Additionally, whether there are tissue-resident immune cells in the four stomachs or increased immune cells originating from the proliferation of tissue-resident immune cells or the infiltration and proliferation of peripheral immune cells remains unknown. We performed homologous gene transformation and utilized a ligand/receptor database specific to mice; however, some cell-to-cell communication information was missing because of incomplete gene transformation. The existing databases cover only a limited number of ligand-binding sites and lack the objective validation with specificity for sheep. Although we identified the cell types of both mucosal epithelium and submucosal layer, the spatial location of these cells still remains speculative, especially non-epithelial cells need further investigation. Further studies that combine multiple datasets, such as spatial transcriptome, in four-chambered stomach model may provide a more definitive test of our model.

## Conclusions

**Fig. 7:**
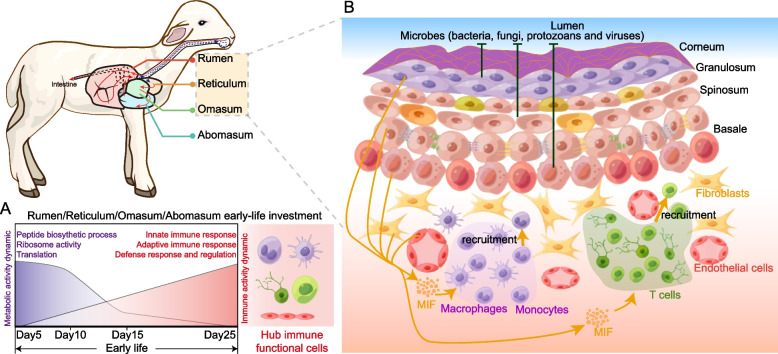
Schematic model summarizing the early-life immune investment in lamb forestomachs and abomasum at both genomic and cellular perspective. **A** Early-life functional investment in lamb rumen, reticulum, omasum and abomasum from d 5 to 25. **B** The cellular interaction and their immune function in lamb forestomachs. This graphic were created using Figdraw (www.figdraw.com)

In summary, we revealed the dynamics of immune functions in lamb’s forestomachs and abomasum at both gene expression and cellular perspective (Fig. 7). Our results indicated that early-life functional investment of four-chambered stomach priorities on immune and defense responses, which are dependent on T cells, monocytes/macrophages, and endothelial cells. Further, the non-immune cells can recruit immune cells through MIF signaling in the forestomachs. Overall, these genomic expression patterns and cellular functions provided novel and comprehensive understanding of early immune development, which facilitates to develop early life nutritional manipulation and health management strategies based on specific targets including key receptors and signaling pathways.

### Supplementary Information

**Additional file 1: Table S1.** The marker genes of different cells. **Table S2.** Summary of marker genes of each cell subtype in rumen. **Table S3.** Summary of marker genes of each cell subtype in reticulum. **Table S4.** Summary of marker genes of each cell subtype in omasum. **Table S5.** Summary of marker genes of each cell subtype in abomasum.

**Additional file 2: Fig. S1.** Top KEGG pathways enriched in the Mred, Mpink, Mmagenta, and Mturquoise. **A–D** Top KEGG pathway enriched in the Mred (**A**), Mpink (**B**), Mmagenta (**C**), and Mturquoise (**D**) (Benjamini-Hochberg corrected *P* value < 0.05). **Fig. S2.** Expression of marker genes of each cell type in four stomachs. **A–D** Dot plot showing the expression of the maker genes of different cell types in the rumen (**A**), reticulum (**B**), omasum (**C**), and abomasum (**D**). **Fig. S3.** Expression of pattern recognition receptors (PPRs) in each cell type in four stomachs. **A**–**D** Dot plot showing the expression of the pattern recognition receptor (PPR) genes including 10 toll-like receptors (TLRs), 11 NOD-like receptors (NLRs), 2 RIG-I-like receptors (RLRs) in different cells in the rumen (**A**), reticulum (**B**), omasum (**C**), and abomasum (**D**). **Fig. S4.** Expression of G-protein-coupled receptors (GPCRs) in each cell type in four stomachs. **A–D** Dot plot showing the expression of the G**-**protein-coupled receptor (GPCR) genes in different cells in the rumen (**A**), reticulum (**B**), omasum (**C**), and abomasum (**D**). **Fig. S5.** H&E-stained image used for histology of the rumen tissue. Solid colored lines were used to separate different layers.

## Data Availability

The RNA-seq and scRNA-seq raw data have been deposited at NCBI Gene Expression Omnibus database (RNA-seq: GSE227043, GSE200295; scRNA-seq: GSE188811). The current study did not generate new code, all codes used to analysis in this study can be found according to corresponding reference.

## Abbreviations

BC: Basal cells
BC**-**PC: Basal or proliferative cells
CC: Chief cells
CPM: Counts per million mapped reads
DEGs: Differentially expressed genes
EndoC: Endothelial cells
EnteC: Enteroendocrine cells
FC: Fibroblasts
GC: Granule cells
GMC: Gland mucus cells
GO: Gene Ontology
GSEA: Gene set enrichment analysis
GSVA: Gene set variation analysis
KEGG: Kyoto Encyclopedia of Genes and Genomes
Mblack: Black module
Mblue: Blue module
Mbrown: Brown module
MC: Monocytes/macrophages
Mgreen: Green module
MIF: Macrophage migration inhibitory factor
Mmagenta: Magenta module
Mpink: Pink module
Mpurple: Purple module
Mred: Red module
Mturquoise: Turquoise module
Myellow: Yellow module
NLRs: NOD-like receptors
ParC: Parietal cells
PC: Proliferative cells
PC1: First principal component
PMC: Pit mucus cells
PPRs: Pattern recognition receptors
RLRs: RIG-I-like receptors
SC: Spinous cells
scRNA-seq: Single-cell RNA sequencing
SMC: Smooth muscle cells
TLRs: Toll-like receptors
UEC: Undefined epithelial cells
WGCNA: Weighted correlation network analysis
