# Supplementary material for: The early life immune dynamics and cellular drivers at single-cell resolution in lamb forestomachs and abomasum
Source: J Anim Sci Biotechnol. 2023 Oct 12;14:130. doi: 10.1186/s40104-023-00933-1 (PMC10568933; doi:10.1186/s40104-023-00933-1)
Supplement: Supplementary file 1 — Additional file 1: Table S1. The marker genes of different cells. Table S2. Summary of marker genes of each cell subtype in rumen. Table S3. Summary of marker genes of each cell subtype in reticulum. Table S4. Summary of marker genes of each cell subtype in omasum. Table S5. Summary of marker genes of each cell subtype in abomasum. [file 40104_2023_933_MOESM1_ESM.docx]

**Table S1** The marker genes of different cells

| **Cell type** | **Marker genes** | **Reference** |
| --- | --- | --- |
| Basal cells | *LOC101117682* (*KRT15*), *DLK2*, *COL17A1*, *LOC101118459* (*KRT14*) | Deprez et al. [1]; Wang et al. [2]; Prenzler et al. [3] |
| Granule cells | *KRT6A*, *S100A8*, *IVL*, *TGM3* | Wang et al. [2]; He et al. [4] |
| Spinous cells | Basal cells^-^ Granule cells^+^ |  |
| Proliferative cells | *CENPF*, *MKI67*, *TOP2A*, *UBE2C* | Shahid et al. [5]; Zhang et al. [6]; He et al. [4] |
| Pit mucus cells | *TFF1*, *GKN1*, *GKN2* | Busslinger et al. [7]; Zhang et al. [6] |
| Gland mucus cells | *MUC6*, *TFF2* | Zhang et al. [6] |
| Chief cells | *LOC101105864* (*PGA*), *CYM*, *BHLHA15* | Zhang et al. [6]; Wu et al. [8]; Deng et al. [9] |
| Parietal cells | *ATP4A*, *ATP4B*, *CBLIF*, *CD24* | Zhang et al. [6]; Kietzmann et al. [10] |
| Fibroblasts | *COL1A2*, *COL3A1*, *COL6A1*, *DCN* | Zhang et al. [6]; Zou et al. [11] |
| Endothelial cells | *PECAM1*, *CDH5*, *VWF*, *ENG* | Zhang et al. [6] |
| Enteroendocrine cells | *CHGA*, *GHRL*, *CPE*, *SCG3* | Wang et al. [12]; Zeve et al. [13] |
| Smooth muscle cells | *ACTA2*, *TAGLN*, *CNN1*, *MYH11* | Zhang et al. [6]; Busslinger et al. [7] |
| Monocytes | *FCGR3A* | Zhang et al. [6] |
| Macrophages | *CSF1R*, *C1QA*, *C1QC* | Zhang et al. [6]; Busslinger et al. [7]; Yuan et al. [14] |
| T cells | *CD3D*, *CD3E*, *CD3G*, *CD69* | Zhang et al. [6] |

**Table S2** Summary of marker genes of each cell subtype in rumen

| **Cluster** | **Cell type** | **Cell subtype** | **Cell subtype marker genes** |
| --- | --- | --- | --- |
| 5 | Proliferative cells | PC | *CENPF, TOP2A, TPX2, MKI67* |
| 0 | Basal cells | BC1 | *MKI67, PCLAF, CENPF, TOP2A* |
| 1 |  | BC2 | *LOC101117657, SLC16A1, LOC101117682, LOC101104808* |
| 3 |  | BC3 | *LOC101114075, CA12, LOC101106610, HMGCS2* |
| 4 |  | BC4 | *CCDC80, IGFBP2, NQO2, FXYD6* |
| 6 |  | BC5 | *MCM4, PCNA, MCM5, MCM3* |
| 7 |  | BC6 | *FOS, JUNB, JUND, SOCS3* |
| 8 |  | BC7 | *FOS, GADD45A, GADD45B, NFKBIZ* |
| 12 |  | BC8 | *RPS5, RACK1, RPS8, CCDC80* |
| 9 | Spinous cells | SC1 | *S100A9, S100A8, LOC101103771, LOC101110777* |
| 10 |  | SC2 | *LAMP1^-^, CLTC^-^, ZBTB7A^-^, EIF5B^-^* |
| 11 |  | SC3 | *RRAD, GSTA1, LOC114108656, FABP4* |
| 2 | Granular cells | GC1 | *KRT6A, LOC101111178, SPINK5, LYPD3* |
| 15 |  | GC2 | *LOC101110777, LOC114113221, LOC114116898, A2ML1* |
| 18 |  | GC3 | *SRGN, SDS, CXCL8, LOC101114790* |
| 13 | Endothelial cells | EndoC | *VIM, COL4A1, CLEC3B, PECAM1* |
| 14 | Fibroblasts | FC | *LGALS1, SPARC, PTGDS, COL1A1* |
| 17 | T cells | TC | *SRGN, LAPTM5, BTG1, LOC101117112* |
| 16 | Monocytes/Macrophages | MC | *DQB, LOC101109492, CD74, LOC101109747* |

PC: Proliferative cells; BC: Basal cells; SC: Spinous cells; GC: Granule cells; EndoC: Edothelial cells; FC: Fibroblasts; TC: T cells; MC: Monocytes/Macrophages

**Table S3** Summary of marker genes of each cell subtype in reticulum

| **Cluster** | **Cell type** | **Cell subtype** | **Cell subtype marker genes** |
| --- | --- | --- | --- |
| 4 | Proliferative cells | PC1 | *TOP2A, CENPF, HMGB2, MKI67* |
| 12 |  | PC2 | *HMGB2, CKS2, CENPF, UBE2C* |
| 0 | Basal cells | BC1 | *COIII, ND4, ATP6, CYTB* |
| 1 |  | BC2 | - |
| 2 |  | BC3 | *FOS, CEBPD, ATF3, GADD45A* |
| 3 |  | BC4 | *CA1, FABP5, FABP4,* |
| 7 |  | BC5 | *UBA52, EEF1D, FXYD3, DLK2* |
| 13 |  | BC6 | *TAGLN, TPM2, MGP, TPM1* |
| 8 | Basal/Proliferative cells | BC-PC | *COX1, TOP2A, CYTB, ND2* |
| 5 | Spinous cells | SC1 | *KRT6A, LOC101102105, SPINK5, GJA1* |
| 11 |  | SC2 | *KRT6A, GSTA1, LOC101102105, SPINK5* |
| 10 | Granular cells | GC | *LOC101102105, SFN, GSTA1, KRTDAP* |
| 6 | Endothelial cells | EndoC1 | *CLEC3B, PLVAP, PECAM1, VIM* |
| 14 |  | EndoC2 | *PECAM1, CLEC3B, VIM, SLC9A3R2* |
| 9 | Fibroblasts | FC1 | *COL1A1, COL3A1, COL1A2, SPARC* |
| 18 |  | FC2 | *PTGDS, MARCKS, LOC101115527, COL3A1* |
| 15 | Smooth muscle cells | SMC | *CALD1, TAGLN, IGFBP7, RARRES2* |
| 17 | T cells | TC | *SRGN, LOC101117112, PTPRC, CORO1A* |
| 16 | Monocytes/Macrophages | MC | *LOC101109747, DQB, LOC101109492, CD74* |

-: Not available

PC: Proliferative cells; BC: basal cells; BC-PC: Basal/Proliferative cells; SC: Spinous cells; GC: Granule cells; EndoC: Endothelial cells; FC: Fibroblasts; SMC: Smooth muscle cells; TC: T cells; MC: Monocytes/Macrophages

**Table S4** Summary of marker genes of each cell subtype in omasum

| **Cluster** | **Cell type** | **Cell subtype** | **Cell subtype marker genes** |
| --- | --- | --- | --- |
| 2 | Proliferative cells | PC | *CENPF, MKI67, HMGB2, TOP2A* |
| 12 | Basal/Proliferative cells | BC-PC | *PCNA, UHRF1, LOC114109701, DNMT1* |
| 0 | Basal cells | BC1 | *CEBPD, ATF3, SERTAD1, SOCS3* |
| 1 |  | BC2 | *RPL22, RPL27A, IGFBP2, LOC101117682* |
| 3 |  | BC3 | *FOS, CA3, IGFBP2, SOCS3* |
| 5 |  | BC4 | *AK2, ACADS, CMBL, IGFBP5* |
| 7 |  | BC5 | *CMBL, SLC16A1, LOC114108656, G0S2* |
| 8 |  | BC6 | *MCM5, PCNA, DNAJC9, MCM4* |
| 9 |  | BC7 | *CA1.1, LOC101114075, CA1, LOC101113599* |
| 4 | Spinous cells | SC | *GSTA1, LYPD3, SPINK5, LOC101117431* |
| 10 | Granular cells | GC | *S100A9, LOC101102105, A2ML1, LYPD2* |
| 14 | Fibroblasts | FC | *COL1A1, GSN, COL3A1, SPARC* |
| 15 | Endothelial cells | EndoC | *CLEC3B, VIM, PECAM1, COL4A1* |
| 11 | Undefined epithelial cells | UEC | *BRI3^-^, ITM2B^-^, SNX3^-^,* |
| 6 | T cells | TC | *LOC114108604, B2M, SRGN, LOC114109611* |
| 13 | Monocytes/Macrophages | MC | *CD74, LOC101109747, DQB, LOC101109492* |

PC: Proliferative cells; BC-PC: Basal/Proliferative cells; BC: Basal cells; SC: Spinous cells; GC: Granule cells; FC: Fibroblasts; EndoC: Endothelial cells; UEC: Undefined epithelial cells; TC: T cells; MC: Monocytes/Macrophages

**Table S5** Summary of marker genes of each cell subtype in abomasum

| **Cluster** | **Cell type** | **Cell subtype** | **Cell subtype marker genes** |
| --- | --- | --- | --- |
| 9 | Proliferative cells | PC | *DUT, HMGB1, SMC2, HMGB2* |
| 4 | Chief cells | CC1 | *PDIA2, TENT5C, ERO1B, BHLHA15* |
| 5 |  | CC2 | *PDIA2, ATF3, FOS, LOC105611302* |
| 3 | Gland mucus/Chief cells | GMC-CC | *AGR2, LOC101107171, KCNE3, FKBP11* |
| 0 | Gland mucus cells | GMC1 | *COIII, CYTB, ATP6, ND4* |
| 2 |  | GMC2 | *LOC101107171, CNMD, KRT8, PGC* |
| 1 | Pit mucus cells | PMC1 | *GKN1, LOC114112251, RRBP1, JUN* |
| 11 |  | PMC2 | *KRT8, KRT19, GKN1, GKN2* |
| 6 | Parietal cells | ParC1 | *ATP4A, ATP4B, CBLIF, SLC16A7* |
| 7 |  | ParC2 | *TNNI3, LOC101105179, LOC101117583, LOC101110664* |
| 13 |  | ParC3 | *ATP5MC3, SLC25A4, COX5A, ATP4B* |
| 17 | Endothelial cells | EndoC | *CLEC3B, PECAM1, EGFL7, RAMP2* |
| 8 | Enteroendocrine cells | EnteC1 | *SCG3, CHGA, SCG5, TMSB15B* |
| 10 |  | EnteC2 | *CHGA, SCGN, SCG5, SCG3* |
| 12 | Fibroblasts | FC | *COL1A1, COL3A1, COL1A2, APOE* |
| 14 | Fibroblast/Smooth muscle cells | FC-SMC | *CALD1, TPM2, MYLK, MYH11* |
| 15 | T cells | TC | *LOC101123290, PTPRC, LAPTM5, LOC101117112* |
| 16 | Monocytes/Macrophages | MC | *CD74, DQB, LOC101109747, CTSS* |

PC: Proliferative cells; CC: Chief cell; GMC-CC: Gland mucus/Chief cells; GMC: Gland mucus cells; PMC: Pit mucus cells; ParC: Parietal cells; EndoC: Endothelial cells; EnteC: Enteroendocrine cells; FC: Fibroblasts; FC-SMC: Fibroblast/Smooth muscle cells; TC: T cells; MC: Monocytes/Macrophages

**References**

1. Deprez M, Zaragosi L-E, Truchi M, Becavin C, Ruiz García S, Arguel M-J, et al. A single-cell atlas of the human healthy airways. Am J Respir Crit Care Med. 2020;202:1636–45.

2. Wang S, Drummond ML, Guerrero-Juarez CF, Tarapore E, MacLean AL, Stabell AR, et al. Single cell transcriptomics of human epidermis identifies basal stem cell transition states. Nat Commun. 2020;11:4239.

3. Prenzler F, Fragasso A, Schmitt A, Munz B. Functional analysis of ZFP36 proteins in keratinocytes. Eur J Cell Biol. 2016;95:277–84.

4. He H, Suryawanshi H, Morozov P, Gay-Mimbrera J, Del Duca E, Kim HJ, et al. Single-cell transcriptome analysis of human skin identifies novel fibroblast subpopulation and enrichment of immune subsets in atopic dermatitis. J Allergy Clin Immunol. 2020;145:1615–28.

5. Shahid M, Lee MY, Piplani H, Andres AM, Zhou B, Yeon A, et al. Centromere protein F (CENPF), a microtubule binding protein, modulates cancer metabolism by regulating pyruvate kinase M2 phosphorylation signaling. Cell Cycle. 2018;17:2802–18.

6. Zhang P, Yang M, Zhang Y, Xiao S, Lai X, Tan A, et al. Dissecting the single-cell transcriptome network underlying gastric premalignant lesions and early gastric cancer. Cell Rep. 2019; 27:1934–47.

7. Busslinger GA, Weusten BLA, Bogte A, Begthel H, Brosens LAA, Clevers H. Human gastrointestinal epithelia of the esophagus, stomach, and duodenum resolved at single-cell resolution. Cell Rep. 2021;34:108819.

8. Wu J-J, Zhu S, Gu F, Valencak TG, Liu J-X, Sun H-Z. Cross-tissue single-cell transcriptomic landscape reveals the key cell subtypes and their potential roles in the nutrient absorption and metabolism in dairy cattle. J Adv Res. 2022;37:1–18.

9. Deng Z, Zhu J, Ma Z, Yi Z, Tuo B, Li T, et al. The mechanisms of gastric mucosal injury: focus on initial chief cell loss as a key target. Cell Death Discov. 2023;9:29.

10. Kietzmann L, Guhr SSO, Meyer TN, Ni L, Sachs M, Panzer U, et al. MicroRNA-193a regulates the transdifferentiation of human parietal epithelial cells toward a podocyte phenotype. J Am Soc Nephrol. 2015;26:1389–401.

11. Zou Y, Ye F, Kong Y, Hu X, Deng X, Xie J, et al. The single-cell landscape of intratumoral heterogeneity and the immunosuppressive microenvironment in liver and brain metastases of breast cancer. Adv Sci. 2023;10:e2203699.

12. Wang Y, Song W, Wang J, Wang T, Xiong X, Qi Z, et al. Single-cell transcriptome analysis reveals differential nutrient absorption functions in human intestine. J Experim Med. 2020; 217:e20191130.

13. Zeve D, Stas E, de Sousa Casal J, Mannam P, Qi W, Yin X, et al. Robust differentiation of human enteroendocrine cells from intestinal stem cells. Nat Commun. 2022;13:261.

14. Yuan Y, Sun D-M, Qin T, Mao S-Y, Zhu W-Y, Yin Y-Y, et al. Single-cell transcriptomic landscape of the sheep rumen provides insights into physiological programming development and adaptation of digestive strategies. Zool Res. 2022;43:634–47.
