# Supplementary material for: The early life immune dynamics and cellular drivers at single-cell resolution in lamb forestomachs and abomasum
Source: J Anim Sci Biotechnol. 2023 Oct 12;14:130. doi: 10.1186/s40104-023-00933-1 (PMC10568933; doi:10.1186/s40104-023-00933-1)
Supplement: Supplementary file 2 — Additional file 2: Fig. S1. Top KEGG pathways enriched in the Mred, Mpink, Mmagenta, and Mturquoise. A–D Top KEGG pathway enriched in the Mred (A), Mpink (B), Mmagenta (C), and Mturquoise (D) (Benjamini-Hochberg corrected P value < 0.05). Fig. S2. Expression of marker genes of each cell type in four stomachs. A–D Dot plot showing the expression of the maker genes of different cell types in the rumen (A), reticulum (B), omasum (C), and abomasum (D). Fig. S3. Expression of pattern recognition receptors (PPRs) in each cell type in four stomachs. A–D Dot plot showing the expression of the pattern recognition receptor (PPR) genes including 10 toll-like receptors (TLRs), 11 NOD-like receptors (NLRs), 2 RIG-I-like receptors (RLRs) in different cells in the rumen (A), reticulum (B), omasum (C), and abomasum (D). Fig. S4. Expression of G-protein-coupled receptors (GPCRs) in each cell type in four stomachs. A–D Dot plot showing the expression of the G-protein-coupled receptor (GPCR) genes in different cells in the rumen (A), reticulum (B), omasum (C), and abomasum (D). Fig. S5. H&E-stained image used for histology of the rumen tissue. Solid colored lines were used to separate different layers. [file 40104_2023_933_MOESM2_ESM.pdf]

Fig. S1

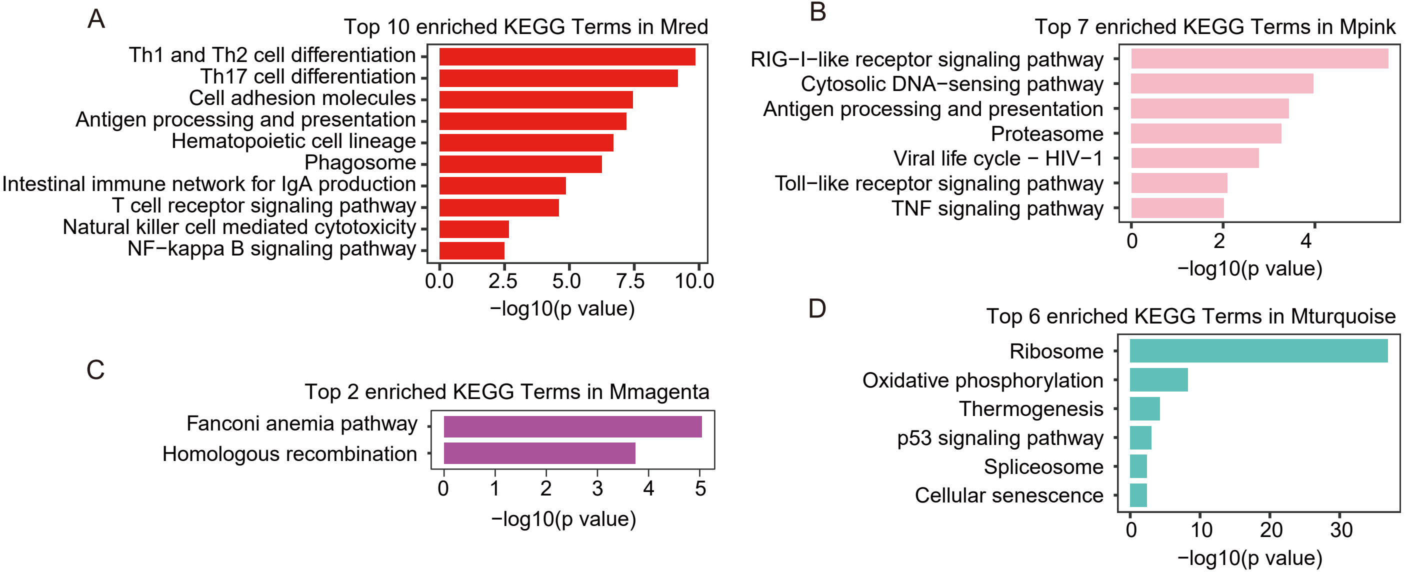
A
Top 10 enriched KEGG Terms in Mred
Th1 and Th2 cell differentiation
Th17 cell differentiation
Cell adhesion molecules
Antigen processing and presentation
Hematopoietic cell lineage
Phagosome
Intestinal immune network for IgA production
T cell receptor signaling pathway
Natural killer cell mediated cytotoxicity
NF−kappa B signaling pathway
0.0
2.5
5.0
7.5
10.0
−log10(p value)
B
Top 7 enriched KEGG Terms in Mpink
RIG−I−like receptor signaling pathway
Cytosolic DNA−sensing pathway
Antigen processing and presentation
Proteasome
Viral life cycle − HIV−1
Toll−like receptor signaling pathway
TNF signaling pathway
0
2
4
−log10(p value)
C
Top 2 enriched KEGG Terms in Mmagenta
Fanconi anemia pathway
Homologous recombination
0
1
2
3
4
5
−log10(p value)
D
Top 6 enriched KEGG Terms in Mturquoise
Ribosome
Oxidative phosphorylation
Thermogenesis
p53 signaling pathway
Spliceosome
Cellular senescence
0
10
20
30
−log10(p value)

Fig. S2

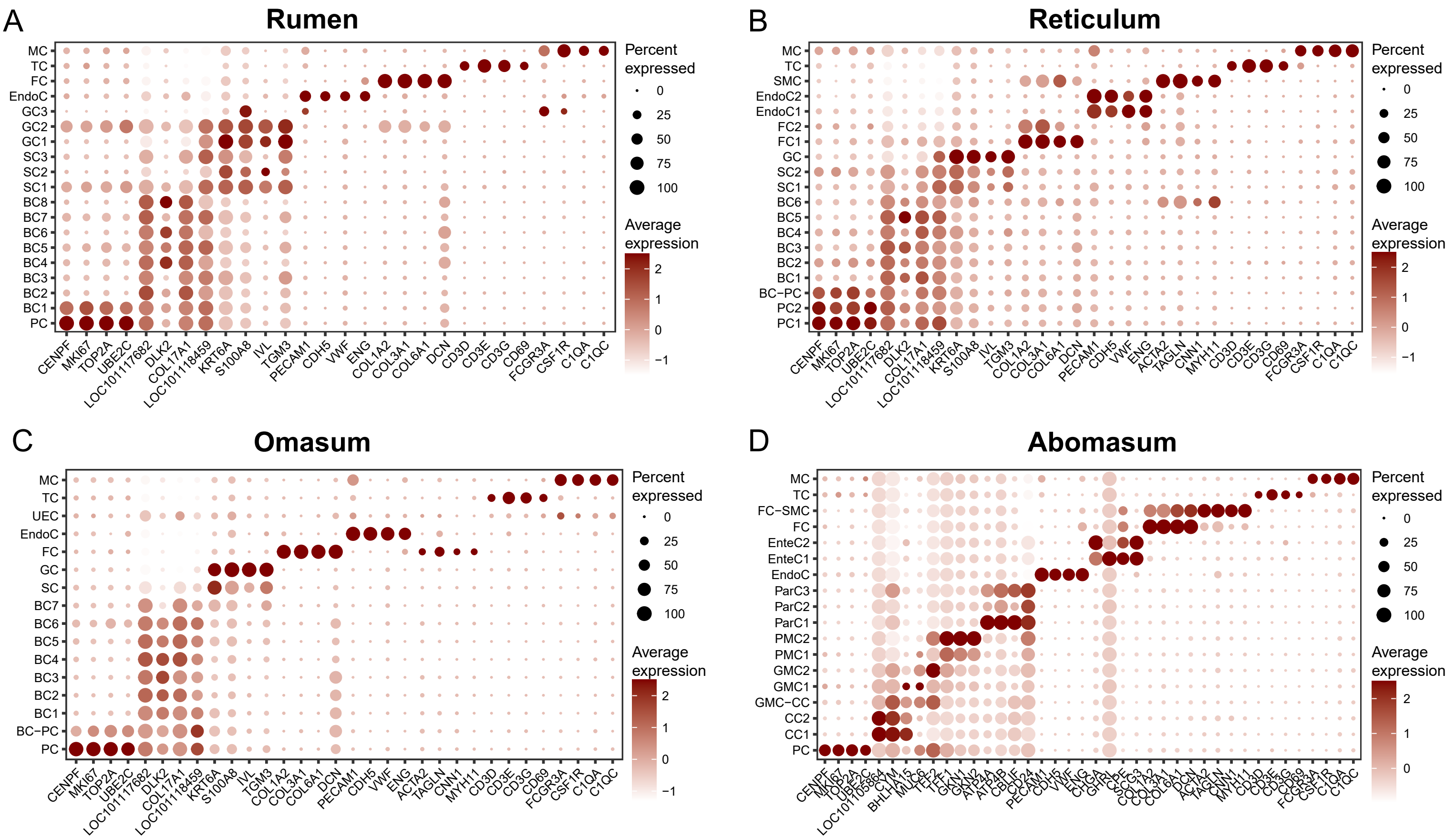

A
Rumen
MC
TC
FC
EndoC
GC3
GC2
GC1
SC3
SC2
SC1
BC8
BC7
BC6
BC5
BC4
BC3
BC2
BC1
PC
CENPF MKI67 TOP2A UBE2C LOC101117682 DLK2 COL17A1 LOC101118459 KRT6A S100A8 IVL TGM3 PECAM1 CDH5 VWF ENG COL1A2 COL3A1 COL6A1 DCN CD3D CD3E CD3G CD69 FCGR3A CSF1R C1QA C1QC
Percent expressed
0
25
50
75
100
Average expression
2
1
0
−1
B
Reticulum
MC
TC
SMC
EndoC2
EndoC1
FC2
FC1
GC
SC2
SC1
BC6
BC5
BC4
BC3
BC2
BC1
BC−PC
PC2
PC1
CENPF MKI67 TOP2A UBE2C LOC101117682 DLK2 COL17A1 LOC101118459 KRT6A S100A8 IVL TGM3 COL1A2 COL3A1 COL6A1 DCN PECAM1 CDH5 VWF ENG ACTA2 TAGLN CNN1 MYH11 CD3D CD3E CD3G CD69 FCGR3A CSF1R C1QA C1QC
Percent expressed
0
25
50
75
100
Average expression
2
1
0
−1
C
Omasum
MC
TC
UEC
EndoC
FC
GC
SC
BC7
BC6
BC5
BC4
BC3
BC2
BC1
BC−PC
PC
CENPF MKI67 TOP2A UBE2C LOC101117682 DLK2 COL17A1 LOC101118459 KRT6A S100A8 IVL TGM3 COL1A2 COL3A1 COL6A1 DCN PECAM1 CDH5 VWF ENG ACTA2 TAGLN CNN1 MYH11 CD3D CD3E CD3G CD69 FCGR3A CSF1R C1QA C1QC
Percent expressed
0
25
50
75
100
Average expression
2
1
0
−1
D
Abomasum
MC
TC
FC−SMC
FC
EnteC2
EnteC1
EndoC
ParC3
ParC2
ParC1
PMC2
PMC1
GMC2
GMC1
GMC−CC
CC2
CC1
PC
CENPF MKI67 TOP2A UBE2C LOC101105864 CYM BHLHA15 MUC6 TFF2 TFF1 GKN1 GKN2 ATP4A ATP4B CBLIF CD24 PECAM1 CDH5 VWF ENG CHGA GHRL CPE SCG3 COL1A2 COL3A1 COL6A1 DCN ACTA2 TAGLN CNN1 MYH11 CD3D CD3E CD3G CD69 FCGR3A CSF1R C1QA C1QC
Percent expressed
0
25
50
75
100
Average expression
2
1
0

Fig. S3

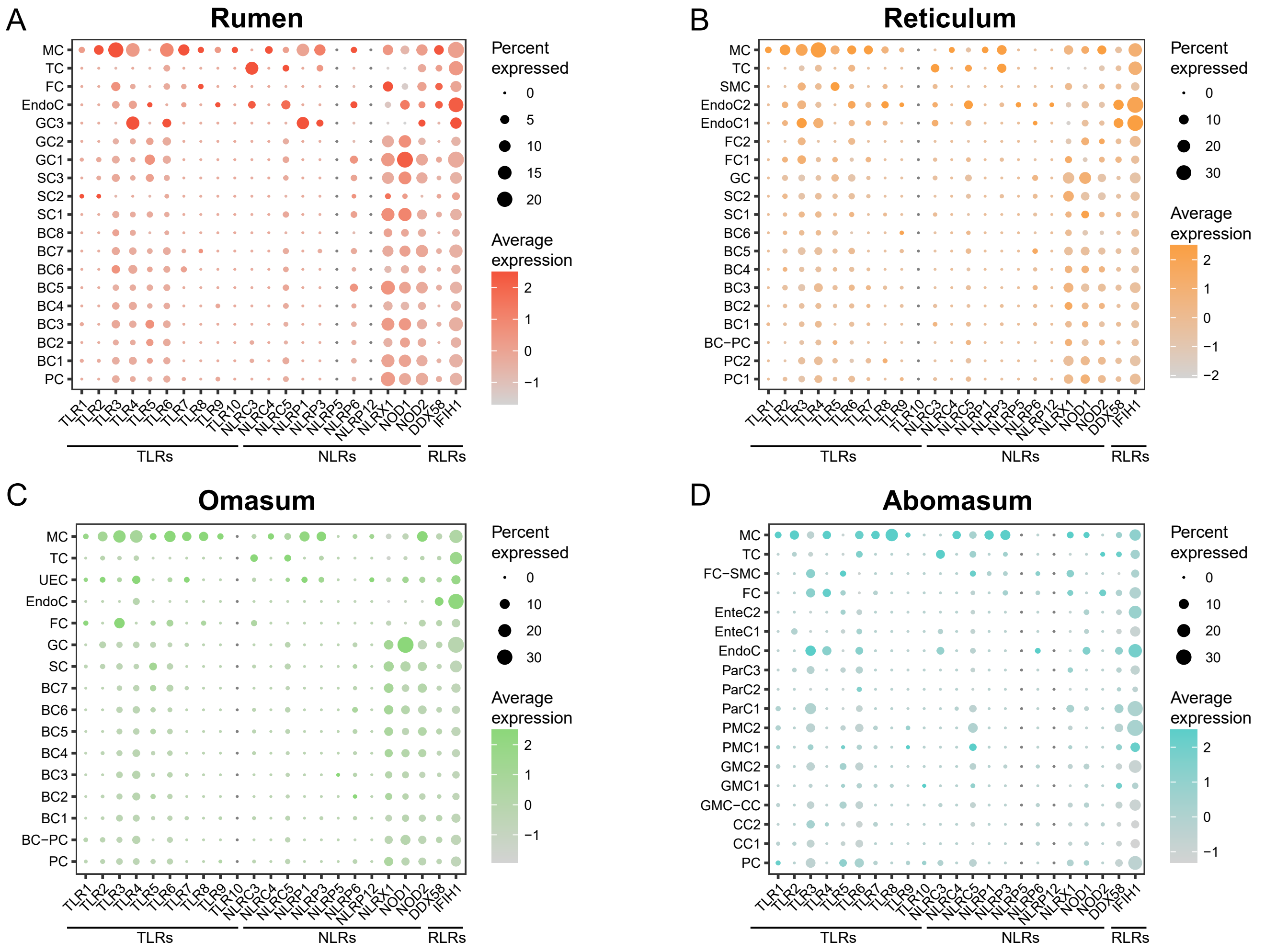
A
Rumen
MC
TC
FC
EndoC
GC3
GC2
GC1
SC3
SC2
SC1
BC8
BC7
BC6
BC5
BC4
BC3
BC2
BC1
PC
TLR1 TLR2 TLR3 TLR4 TLR5 TLR6 TLR7 TLR8 TLR9 TLR10 NLRC3 NLRC4 NLRC5 NLRP1 NLRP3 NLRP5 NLRP6 NLRP12 NLRX1 NOD1 NOD2 DDX58 IFIH1
TLRs
NLRs
RLRs
Percent expressed
0
5
10
15
20
Average expression
2
1
0
−1
B
Reticulum
MC
TC
SMC
EndoC2
EndoC1
FC2
FC1
GC
SC2
SC1
BC6
BC5
BC4
BC3
BC2
BC1
BC−PC
PC2
PC1
TLR1 TLR2 TLR3 TLR4 TLR5 TLR6 TLR7 TLR8 TLR9 TLR10 NLRC3 NLRC4 NLRC5 NLRP1 NLRP3 NLRP5 NLRP6 NLRP12 NLRX1 NOD1 NOD2 DDX58 IFIH1
TLRs
NLRs
RLRs
Percent expressed
0
10
20
30
Average expression
2
1
0
−1
−2
C
Omasum
MC
TC
UEC
EndoC
FC
GC
SC
BC7
BC6
BC5
BC4
BC3
BC2
BC1
BC−PC
PC
TLR1 TLR2 TLR3 TLR4 TLR5 TLR6 TLR7 TLR8 TLR9 TLR10 NLRC3 NLRC4 NLRC5 NLRP1 NLRP3 NLRP5 NLRP6 NLRP12 NLRX1 NOD1 NOD2 DDX58 IFIH1
TLRs
NLRs
RLRs
Percent expressed
0
10
20
30
Average expression
2
1
0
−1
D
Abomasum
MC
TC
FC−SMC
FC
EnteC2
EnteC1
EndoC
ParC3
ParC2
ParC1
PMC2
PMC1
GMC2
GMC1
GMC−CC
CC2
CC1
PC
TLR1 TLR2 TLR3 TLR4 TLR5 TLR6 TLR7 TLR8 TLR9 TLR10 NLRC3 NLRC4 NLRC5 NLRP1 NLRP3 NLRP5 NLRP6 NLRP12 NLRX1 NOD1 NOD2 DDX58 IFIH1
TLRs
NLRs
RLRs
Percent expressed
0
10
20
30
Average expression
2
1
0
−1

Fig. S4

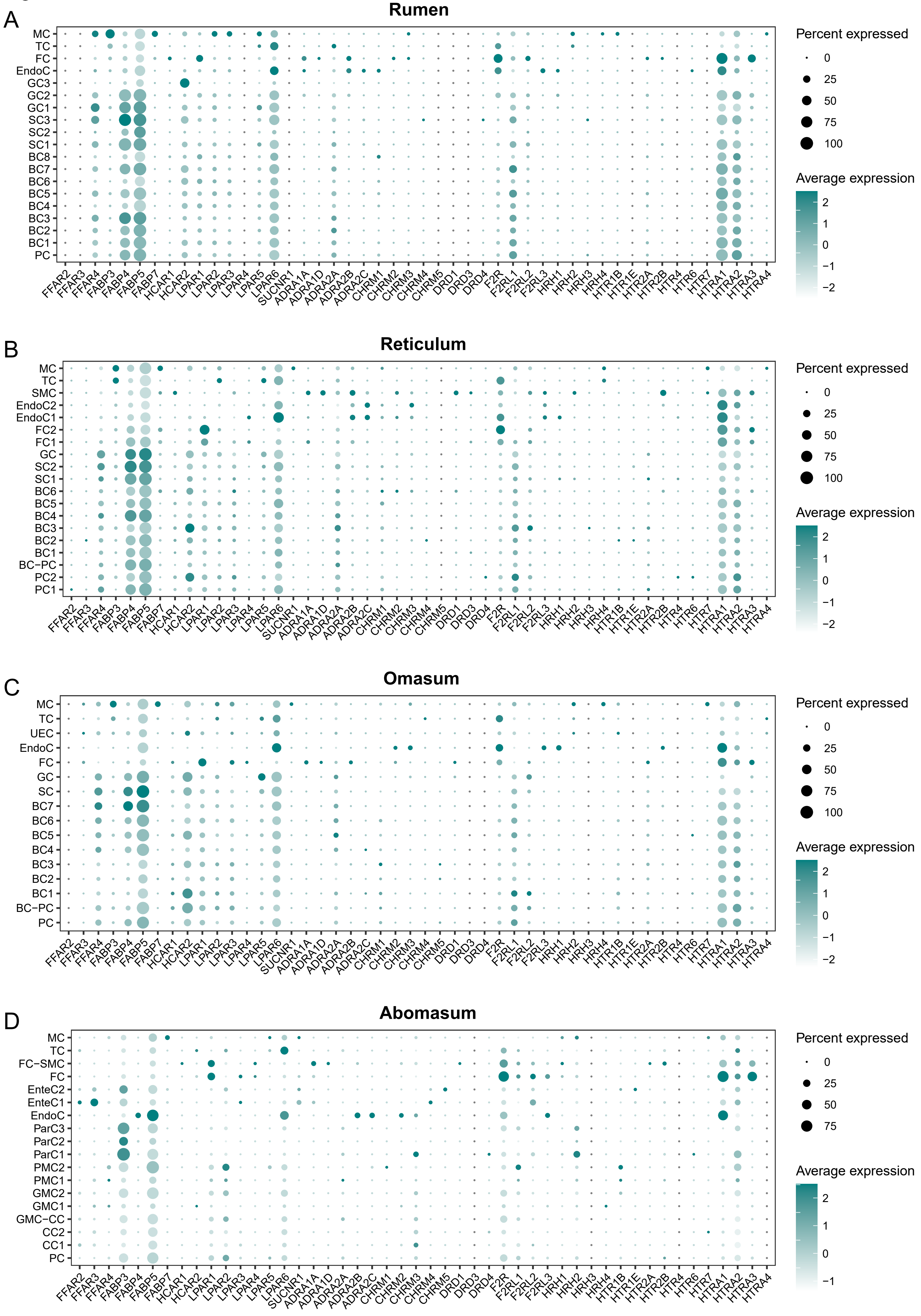
A
Rumen
MC TC FC EndoC GC3 GC2 GC1 SC3 SC2 SC1 BC8 BC7 BC6 BC5 BC4 BC3 BC2 BC1 PC
FFAR2 FFAR3 FFAR4 FABP3 FABP4 FABP5 FABP7 HCAR1 HCAR2 LPAR1 LPAR2 LPAR3 LPAR4 LPAR5 LPAR6 SUCNR1 ADRA1A ADRA1D ADRA2A ADRA2B ADRA2C CHRM1 CHRM2 CHRM3 CHRM4 CHRM5 DRD1 DRD3 DRD4 F2R F2RL1 F2RL2 F2RL3 HRH1 HRH2 HRH3 HRH4 HTR1B HTR1E HTR2A HTR2B HTR4 HTR6 HTR7 HTRA1 HTRA2 HTRA3 HTRA4
Percent expressed
0 25 50 75 100
Average expression
2 1 0 −1 −2
B
Reticulum
MC TC SMC EndoC2 EndoC1 FC2 FC1 GC SC2 SC1 BC6 BC5 BC4 BC3 BC2 BC1 BC−PC PC2 PC1
FFAR2 FFAR3 FFAR4 FABP3 FABP4 FABP5 FABP7 HCAR1 HCAR2 LPAR1 LPAR2 LPAR3 LPAR4 LPAR5 LPAR6 SUCNR1 ADRA1A ADRA1D ADRA2A ADRA2B ADRA2C CHRM1 CHRM2 CHRM3 CHRM4 CHRM5 DRD1 DRD3 DRD4 F2R F2RL1 F2RL2 F2RL3 HRH1 HRH2 HRH3 HRH4 HTR1B HTR1E HTR2A HTR2B HTR4 HTR6 HTR7 HTRA1 HTRA2 HTRA3 HTRA4
Percent expressed
0 25 50 75 100
Average expression
2 1 0 −1 −2
C
Omasum
MC TC UEC EndoC FC GC SC BC7 BC6 BC5 BC4 BC3 BC2 BC1 BC−PC PC
FFAR2 FFAR3 FFAR4 FABP3 FABP4 FABP5 FABP7 HCAR1 HCAR2 LPAR1 LPAR2 LPAR3 LPAR4 LPAR5 LPAR6 SUCNR1 ADRA1A ADRA1D ADRA2A ADRA2B ADRA2C CHRM1 CHRM2 CHRM3 CHRM4 CHRM5 DRD1 DRD3 DRD4 F2R F2RL1 F2RL2 F2RL3 HRH1 HRH2 HRH3 HRH4 HTR1B HTR1E HTR2A HTR2B HTR4 HTR6 HTR7 HTRA1 HTRA2 HTRA3 HTRA4
Percent expressed
0 25 50 75 100
Average expression
2 1 0 −1 −2
D
Abomasum
MC TC FC−SMC FC EnteC2 EnteC1 EndoC ParC3 ParC2 ParC1 PMC2 PMC1 GMC2 GMC1 GMC−CC CC2 CC1 PC
FFAR2 FFAR3 FFAR4 FABP3 FABP4 FABP5 FABP7 HCAR1 HCAR2 LPAR1 LPAR2 LPAR3 LPAR4 LPAR5 LPAR6 SUCNR1 ADRA1A ADRA1D ADRA2A ADRA2B ADRA2C CHRM1 CHRM2 CHRM3 CHRM4 CHRM5 DRD1 DRD3 DRD4 F2R F2RL1 F2RL2 F2RL3 HRH1 HRH2 HRH3 HRH4 HTR1B HTR1E HTR2A HTR2B HTR4 HTR6 HTR7 HTRA1 HTRA2 HTRA3 HTRA4
Percent expressed
0 25 50 75
Average expression
2 1 0 −1

Fig. S5

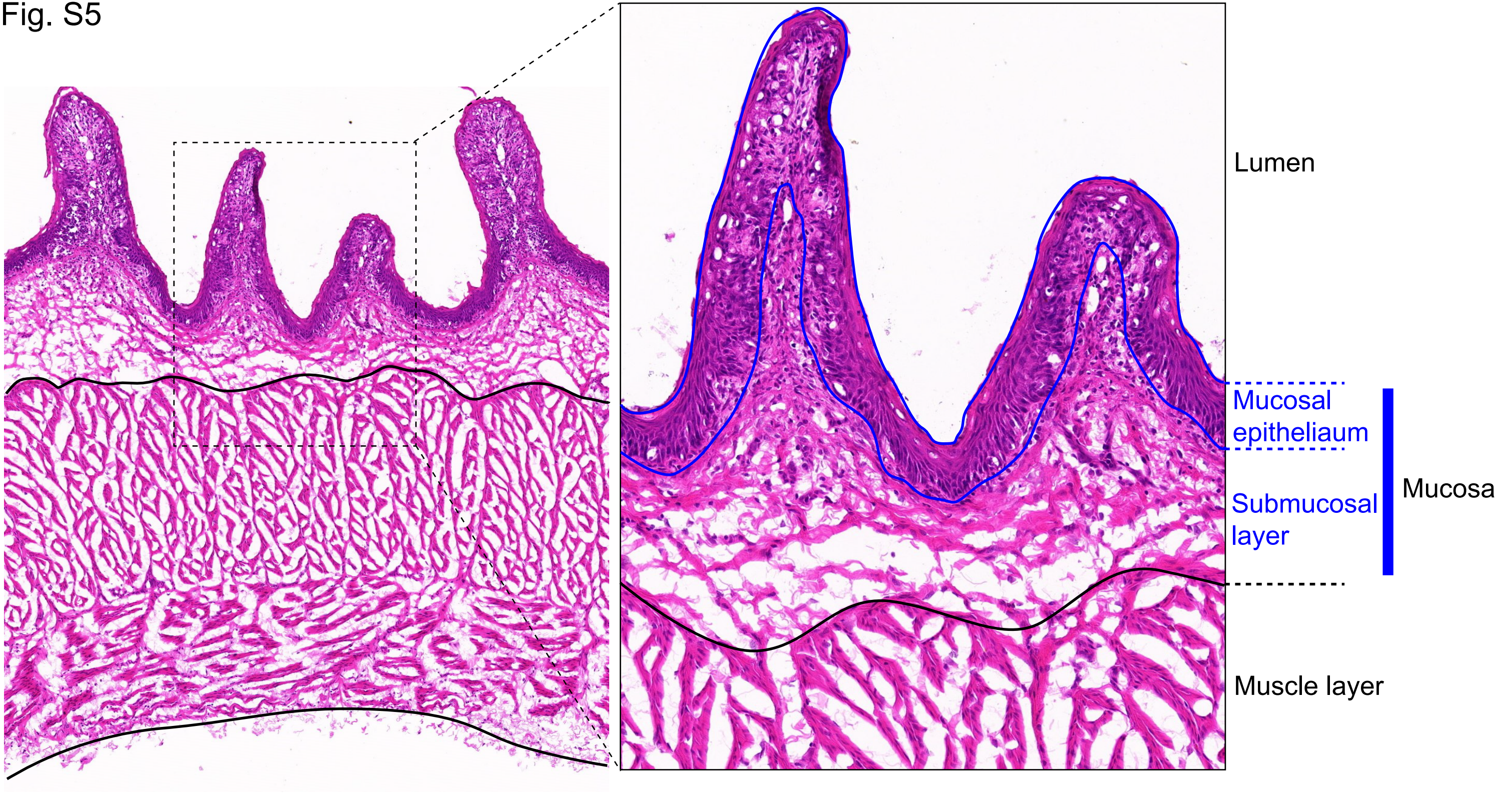
Lumen
Mucosal
epitheliaum
Submucosal
layer
Mucosa
Muscle layer
